# Predator Cues Induce Defensive Responses in Marine Prey: A Metabolomics Decoding of Blue Crab Urine Biomarkers

**DOI:** 10.1021/acs.jproteome.6c00084

**Published:** 2026-08-28

**Authors:** Marisa R. Cepeda, Benton R. Jaco, Benjamin A. Belgrad, Maria E. Branch, Samuel G. Moore, Sarah H. Roney, David A. Gaul, Delbert L. Smee, Marc J. Weissburg, Julia Kubanek

**Affiliations:** † School of Chemistry and Biochemistry, 1372 Georgia Institute of Technology, Atlanta, Georgia 30332, United States; ‡ School of Biological Sciences, 1372 Georgia Institute of Technology, Atlanta, Georgia 30332, United States; § Dauphin Island Sea Lab, Dauphin Island, Alabama 36528, United States; ∥ Parker H. Petit Institute for Bioengineering and Bioscience, Georgia Institute of Technology, Atlanta, Georgia 30332, United States; ⊥ School of Marine and Environmental Sciences, University of South Alabama, Mobile, Alabama 36608, United States

**Keywords:** urine, metabolomics, biomarker, chemical cue, predator−prey, Atlantic blue crab, eastern oyster, mass spectrometry

## Abstract

Prey rely on chemical cues to detect predators, especially in coastal ecosystems where danger is concealed by turbid waters. Juvenile oysters (*Crassostrea virginica*) respond to blue crab (*Callinectus sapidus*) chemical cues by growing stronger shells, while mud crabs (*Panopeus herbstii*) reduce foraging to avoid detection. However, the chemical composition of marine fear cues is largely unknown, partly due to methodological challenges. In this study, metabolomics was used to pinpoint predator metabolites that correlated with induced juvenile oyster defenses when exposed to the urine of blue crabs fed either oysters or mud crabs and to determine the chemical composition of these fear cues. Genetic algorithms applied to urine mass spectra coupled with bioassay data confirmed that metabolites from blue crab urine are responsible for fear responses by oysters. Sixteen compounds (of over 700) were determined to be potentially important cues used by juvenile oysters to detect blue crabs, with 3-(1-methyl-*1H*-imidazol-4-yl) propanoic acid identified as the most important contributor to this effect. These results indicate that oysters rely on certain metabolites that are ubiquitous in a variety of chemical blends to evaluate predation risk, and the response of juvenile oysters is not dependent on a singular metabolite but on numerous metabolites working in conjunction.

## Graphical abstract

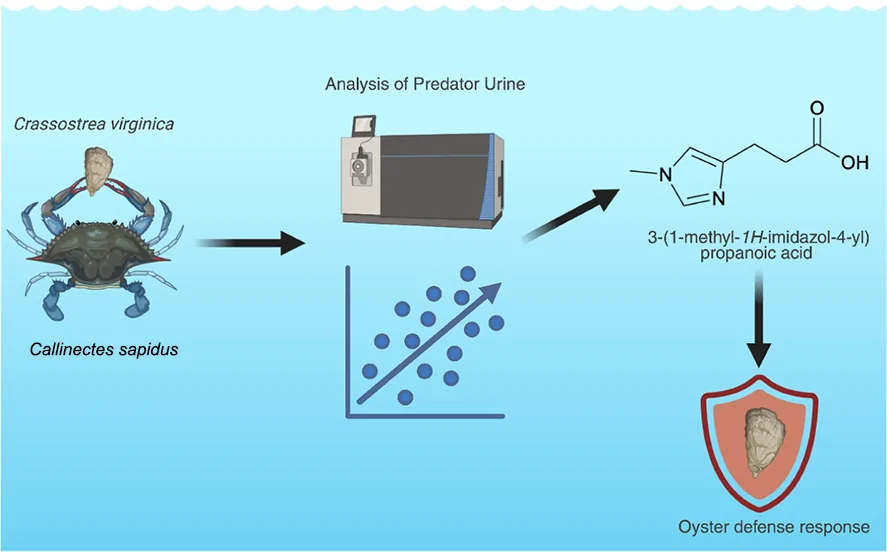

## 1 Introduction

Detection of, and response to, chemical cues increase the fitness of many organisms, allowing predators to find prey and prey to avoid predation; each party leveraging chemical information from the environment for their benefit. Predators use chemicals to locate a potential food source, and rely on taste and smell to determine whether it is palatable or safe to eat.^1^ At the same time, prey assess predation risk by detecting molecules released by predators, conspecifics, and other neighboring species within a shared habitat.^2^ For instance, the “smell of fear” can be a mixture of exudates from injured prey and metabolic waste released by feeding predators:^2^ together these cues affect behavior,^3^ physiology,^4^ morphology, and life history. Such cues significantly impact community structure and ecosystem function via nonconsumptive effects of predation, not only the effects of direct removal of prey by predators.

Information contained in chemical cues is conveyed by a complex language comprised of numerous potential molecules. Traditionally, studying these messenger molecules involves multiple rounds of different chromatographic separations coupled with repeated measurements of physiological, morphological, or behavioral responses, painstakingly leading to pure compounds responsible for a biological effect.^5,6^ This method, known as bioassay-guided fractionation, has proven useful in many instances, resulting in a plethora of natural products and chemical ecology discoveries, but it is not consistently successful in identifying the components required for biological activity.^7^ Biological activity of putative cues isolated from organisms often is reduced or lost after several separation steps. Possible reasons include chemical instability, inefficient recovery following separation, or the need for multiple components to be present in specific ratios.^8^

Metabolomics is a modern approach for studying these molecular messages, relying on chemical profiling coupled with statistical analysis to identify metabolites of potential interest. Comparative metabolomic profiling allows for the identification of chemical features associated with dramatic or subtle differences between samples of differing biological activity.^5,8^ Minimal sample preparation and not needing to conduct multiple rounds of separations and bioassays increases efficiency in comparison to the more traditional route of bioassay-guided fractionation, especially if the accompanying bioassays are slow, labor-intensive, and consume substantial quantities of chemical cue. Chemical features represented in multivariate statistical models can be traced to MS^2^ fragmentation fingerprints of specific molecular structures and substructures using online databases, supporting compound annotation. If bioactive molecules are previously unknown metabolites, they may be isolated and characterized using a combination of spectroscopic techniques. In the past decade, metabolomics has provided a platform for generating chemical profiles of many marine organisms, such as corals,^9^ sponges,^10^ algae,^11,12^ and bivalves.^13,14^ It has also been successfully applied to discover a diatom sex pheromone^15^ and the effects of allelopathic phytoplankton competition;^16^ however, only a few studies have identified chemical cues that mediate perception of predators by their prey.^17–20^

Urine is an important cue source, rich in small organic molecules known to influence animal behavior, particularly prey responses to predators.^21^ The water-soluble and volatile properties of urinary metabolites facilitate transport from emitter to recipient. Given that many animals urinate frequently, it is advantageous for prey to use predator urine for assessing predation risk. Identifying urinary cues has been relatively more successful in terrestrial than in marine systems where urine can be harvested directly from the animal.^21^ A unique challenge for marine systems is that waterborne molecules released by aquatic predators are difficult to isolate from seawater due to low natural concentrations in a high salt matrix.^22^ However, urine can be directly sampled from some marine animals, including crustaceans and fish, allowing analysis of its chemical composition without interference from the surrounding environment using techniques such as those developed for metabolomics.^18,23,24^

Commonalities exist among urinary metabolites of different species,^25^ but the majority of chemical characterization efforts have focused on human urine,^26,27^ especially in relation to aspects such as diet,^28^ age, and sex, all of which affect its composition.^29^ Individual variation and chemical complexity can potentially impact urine potency as a chemical cue, as demonstrated by studies relating urine of different predator diets to danger perceived by prey.^18,30^ The specific cue combinations and unique ratios necessary to accurately convey predation risk are unknown, despite these being key factors that determine the uniqueness of a particular scent.^31,32^ These studies have raised important questions about how prey reliably decode risk. Diet-specific metabolites are strong signals that a predator has eaten a conspecific and comprises a threat. However, prey also respond to generalist predators regardless of diet.^33,34^

The present study identifies molecules in blue crab urine, after having consumed either oysters or mud crabs, that are perceived by juvenile oyster prey as threatening and capitalizes on a situational advantage where it is possible to acquire urine directly from predators, thereby avoiding complications associated with analyzing molecules in dilute seawater. It further leverages genetic algorithms applied to metabolomics data sets and multivariate statistics to identify blue crab urine biomarkers indicative of predation risk to oysters. Previous work identified homarine (**1**) and trigonelline (**2**) as two urine constituents important for oysters in perceiving predation risk; however it was evident in those studies that these are not the only cues used to detect predatory blue crabs.^18,36^ The current study provides insights about the commonalities and differences between fear cue blends originating from a common predator, emphasizing the complexity of these cues, and how they are influenced by diet, especially in the context of predator–prey interactions.

## 2 Materials and Methods

To identify chemical cues that evoke fear responses in prey, we conducted morphological assays using live prey with metabolomic analyses of predator urine samples that spanned a range of fear-inducing effects, seeking correlation between potency in bioassays and chemical composition. Institutional Animal Care and Use Committee (IACUC) approval was not required for this study because only invertebrate animals (crabs and oysters) were used, in accordance with institutional requirements.

### 2.1 Blue Crab Urine Preparation

Blue crabs (*Callinectus sapidus*) were caught in commercial crab traps placed in Mobile Bay sand housed in individual tanks with natural seawater. Water was drawn offshore from Mobile Bay, AL, USA, filtered (200 μm mesh), and allowed to settle in a tank for 3 days to remove particulates before use. Seawater salinity was maintained at 20 ± 2 ppt throughout all experiments. Crabs were starved for two to 3 days prior to being fed either oysters (*Crassostrea virginica*) or mud crabs (*Panopeus herbstii*) once every 2 days. All blue crabs were maintained on this diet for 1 week before beginning urine collection, which was performed twice per week. Crabs were chilled on ice to quiescence prior to siphoning urine from the nephropore using a 23 gauge-needle and light vacuum suction.^36^

Urine collected from individual crabs was combined into mixtures to provide enough urine for both the oyster shell strengthening and mud crab feeding assays (∼36 mL/mixture). Urine samples from individual blue crabs were stored at −80 °C before being combined, ensuring that the smallest number of crabs contributed to each mixture. This maximized the chemical diversity of the urine samples, as desired for metabolomics analysis. For the current study, each mixture was composed of urine from 3 to 12 individual blue crabs, constituting one biological replicate. The same 24 urine mixtures (n = 12 mud crab diet and n = 12 oyster diet) were used for both bioassays, enabling direct comparison of chemical features associated with each biological response.

### 2.2 Initial Oyster Shell Strengthening Assay

The Auburn University Shellfish Laboratory provided diploid oyster larvae in two spawns in early May and July 2020 that were used in the bioassay performed at Dauphin Island Sea Lab (DISL) in Dauphin Island, AL. This allowed bioassays to be conducted in two blocks between May and September of the same year.

Oysters were settled onto 4.5 cm × 4.5 cm marble tiles and allowed to grow for 1 week in 1250 L mesocosms with flow-through seawater prior to experimentation. Oyster spat were then transferred to aquaria and repeatedly exposed to 1 mL mixtures of blue crab urine from crabs that subsisted on either an oyster or mud crab diet (n = 3 mixtures in block 1 and n = 9 mixtures in block 2 per predator diet), settled seawater (negative control; n = 3 mixtures in block 1 and n = 9 mixtures in block 2), or a positive control of predator water (0.5 L water from a tank housing blue crabs fed oysters added to 1.5 L settled seawater; n = 3 in block 1 and n = 7 in block 2). All seawater was collected and maintained under the same conditions described in Section 2.1. Aquaria were distributed within three flow-through tanks that provided a water bath to keep aquaria temperatures the same as ambient seawater temperatures. Aquaria water was changed and then cues were added biweekly for approximately 8 weeks. After this period, either 9 (block 1) or 20 (block 2) oyster spat were crushed from each biological replicate to assess shell strength. Oyster shell length was measured with digital calipers, and the force required to crush a shell (N) with a Kistler 9207 force sensor coupled to a 5995 charge amplifier. Crushing force was divided by shell length to produce a size-standardized metric of shell strength (i.e., standardized crushing force, N/mm) because larger individuals naturally have a stronger shell as a byproduct of their size. These values were averaged for oysters within the same aquaria to produce a measure of urine mixture potency used in the below metabolomic analyses (Figure 1).

**Figure 1:**
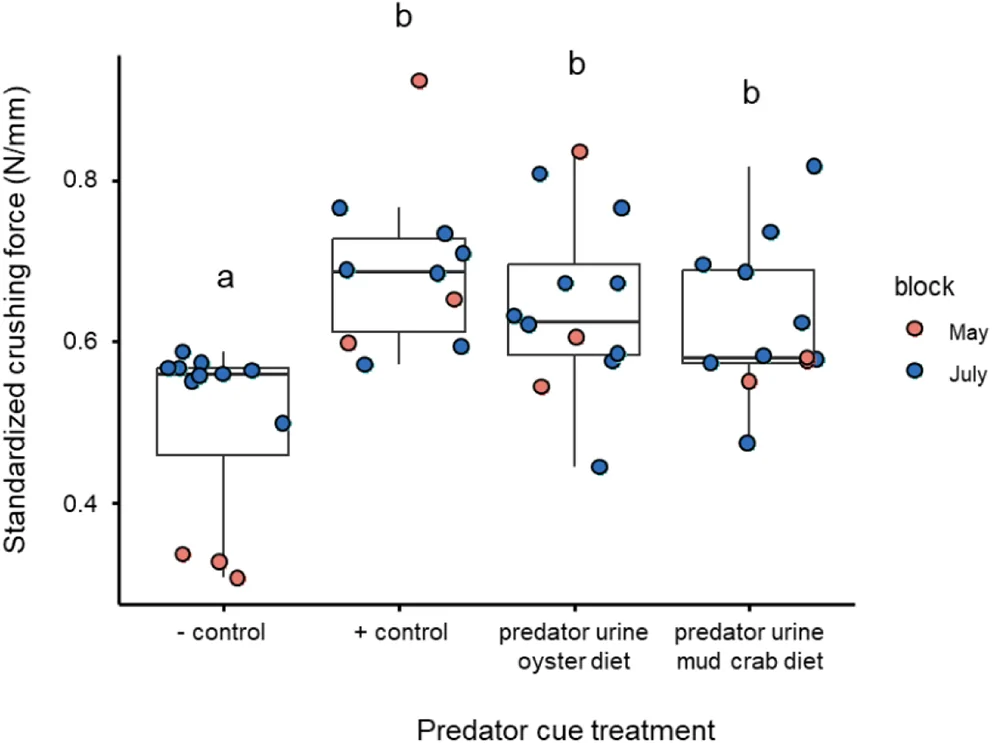
Effects on juvenile oyster shell strength for different predator cue solutions (- control = seawater, + control = water from a tank housing blue crabs, mixtures of urine from blue crabs fed oysters, mixtures of urine from blue crabs fed mud crabs). Circles represent average shell strength for a particular cue mixture for that treatment (*n* = 9; 20 individuals). Oysters were reared in two experimental blocks either in May (red circles, *n* = 3 mixtures) or July (blue circles, *n* = 7; 9 mixtures). Boxes indicate the 25th and 75th percentiles, whiskers denote data points no farther than 1.5x interquartile range and the median is shown by the horizontal line within each box. Distinct letters above each box denote significant differences between treatments in line with Compact Letter Display convention.

Shell strength across the different cue treatments was analyzed using a generalized linear mixed model (GLMM) with a gamma distribution. Cue treatment was set as a fixed categorical variable, while holding aquaria nested within the water bath tank were set as random effects to control for nonindependence among individuals. All cue treatments were initially compared to the negative control in the R package “lme4” followed by post hoc pairwise comparisons to determine significantly different groups using the general linear hypotheses function in the R package: multcomp.^37^ Statistical analyses were conducted using R v4.4.2. Additional details for the oyster bioassay are provided in Roney et al.^36^

### 2.3 Blue Crab Urine Chemical Profiling

Aliquots of the same 24 blue crab urine samples used for both bioassays were brought from Dauphin Island Sea Lab to the Georgia Institute of Technology, GA, USA where they were stored at −80 °C. ^1^H nuclear magnetic resonance (NMR) spectroscopy and ultrahigh performance liquid chromatography–mass spectrometry (UHPLC-MS) data sets were acquired for all urine samples at the Georgia Institute of Technology’s NMR Center and Systems Mass Spectrometry Core. These complementary data were used to explore the metabolic composition of blue crab urine.

#### 2.3.1 NMR Metabolomics

Samples were prepared for NMR spectral analysis by combining 138 μL blue crab urine, 46 μL phosphate buffer (0.1 M pH 7.4), and 16 μL 0.1% w/v 3-(trimethylsilyl) propionic-2,2,3,3-d_4_ acid (TMSP). Phosphate buffer and TMSP solutions were made in D_2_O (99.9% atom D_2_O; Cambridge Isotope Laboratories). Solutions were mixed via micropipette and 175 μL were transferred to 3 mm NMR precision tubes (Wilmad-LabGlass). All spectra were acquired using a Bruker Avance IIIHD 800 MHz spectrometer equipped with a 3 mm cryoprobe. For water suppression, the Bruker zgpr pulse sequence was used with a relaxation delay of 10 s. Spectra were centered at 3758.89 Hz, spectral width was set to 13.9 ppm, receiver gain was set to 16, and 256 scans were acquired per sample.

Data were processed in Metabolab.^38^ Spectra were phased, the 3-(Trimethylsilyl)­propionic acid-d_4_ sodium salt (TMSP) reference peak was set to 0 ppm, and spline baseline corrected choosing baseline points spanning the entire spectral width. The TMSP peak, residual artifacts from the suppressed water peak, and regions with a noisy baseline were excluded as they are not relevant for statistical analyses. Spectra were aligned, filtered for noise, and then binned using a width of 0.005 ppm. Probabilistic quotient normalization (PQN) was applied to reduce systematic variation. Processed spectra were imported into PLS Toolbox 9.0 ^39^ where they were log transformed and mean-centered prior to multivariate statistical analyses.

#### 2.3.2 Nontargeted Mass Spectrometry Metabolomics

Blue crab urine was thawed at room temperature and then kept on ice during sample preparation. Pooled urine samples for each diet (mud crab and oyster) were prepared by combining equal volumes of all samples for a single diet. For both pooled and biological replicate urine samples, 20 μL urine was added to 180 μL Liquid Chromatography–Mass Spectrometry (LC-MS) grade methanol, vortex mixed, centrifuged at 19980 × g for 10 min to pellet any precipitate, and the supernatant was transferred to a clean vial.

Analysis was performed using a Vanquish UHPLC coupled to an Orbitrap ID-X Tribrid mass spectrometer (Thermo Fisher Scientific). Chromatographic separation was conducted for 1 μL sample injections using an ACQUITY UPLC BEH Amide column (2.1 × 150 mm, 1.7 μm particle size, Waters Corporation) held at a temperature of 40 °C. Compounds were eluted from the column at 40 mL/min using 80:20 water/acetonitrile with 10 mM ammonium formate and 0.1% formic acid (mobile phase A), and acetonitrile with 0.1% formic acid (mobile phase B) for the following gradient program: 0 min 5% A; 0.5 min 5% A; 8 min 60% A; 9.4 min 60% A; 9.5 min 5% A; 12 min 5% A (Optima, LC-MS grade, Fisher Scientific).

The following settings were used for the heated electrospray ionization (HESI) source: positive ionization mode, a vaporizer temperature of 275 °C, a spray voltage of 3.5 kV, and sheath, auxiliary, and sweep gas flows of 40, 8, and 1 L/h, respectively. For the nontargeted metabolomics experiment, full scan data from 70 to 600 *m*/*z* were acquired with the orbitrap at a resolution of 60,000 followed by data-dependent acquisition (DDA) of tandem mass spectra with the ion trap to ensure accurate mass spectra using both higher energy collisional dissociation (HCD, collision energies 15, 30, and 45 eV) and collision-induced dissociation (CID, collision energy 35 eV). For HCD, precursor ions were isolated in the ion trap using a 0.8 *m*/*z* window and activated at 50%. Data acquisition was performed using Xcalibur Version 4.3.73.11 ^40^ followed by processing in Compound Discoverer 3.3.0.550.^41^ Peak areas were exported from Compound Discoverer following drift correction based on pooled quality control injections and further processed in PLS Toolbox 9.0. The data was processed with and without PQN prior to log transformation and mean centering, generating two base models that would later be used for partial least-squares regression (PLS-R) analyses.

### 2.4 Multivariate Statistical Analyses of Spectroscopic Data and Variable Selection

Both preprocessed ^1^H NMR and LC-MS metabolomics data sets were analyzed in PLS Toolbox 9.0 by principal component analysis (PCA) and partial least-squares discriminant analysis (PLS-DA), in order to test the effect of predator diet on prey response. These spectral data sets then were paired with oyster shell strengthening assay data to build PLS-R models. Unlike for ^1^H NMR spectroscopy, mass spectral features are considered independent variables so genetic algorithm variable selection was used to refine the mass spectrometry feature list by removing unwanted features that did not correlate with bioactivity, greatly improving the cross validation for these models. Different combinations of genetic algorithm parameters were compared to determine which ones resulted in the lowest root mean squared error of cross validation (RMSECV). A 2 × 2 factorial design was implemented to create four models: data processed with or without PQN either with or without a percent of variables target range (5–15% with a penalty slope of 0.05). The following parameters were used for all genetic algorithm runs: a population size of 256, 20% initial terms, 100 max generations, 50% at convergence, 0.005 mutation rate, single crossover, 10 max Latent Variables, venetian blinds cross validation (6 splits and thickness = 1), 2 cross validation iterations, and 5 replicate runs. Although each genetic algorithm tested hundreds of different variable combinations, selected variable lists are those with the lowest RMSECV. All multivariate figures were generated using PLS Toolbox and subsequently reformatted for publication quality visualization in Google Slides (e.g., increased font size and symbol clarity), without alteration of the underlying data. Variable importance parameter (VIP) scores were compared for all features, and those with a score of 0.75 or higher or that were selected by at least two models were compiled into a prioritized list, as these features are the most ideal biomarker candidates. Pearson correlation coefficients were calculated for all features in Microsoft Excel (Microsoft 365). Permutation tests were performed in PLS Toolbox to confirm that all models were robust.

### 2.5 Annotation of Blue Crab Urine Biomarkers via LC-MS/MS Spectra

A follow-up targeted MS/MS experiment was later performed to acquire missing fragmentation spectra of promising MS features arising from PLS-R models, which produced from refined feature lists using genetic algorithm variable selection. This was necessary because MS/MS spectra for some of the selected features were not acquired in the nontargeted experiment. A pooled urine sample was prepared from equal volumes of all 24 urine samples as described previously. The general method parameters were the same as for the nontargeted MS/MS experiment; however, targets were chosen to maximize data collection for each injection. Reducing target overlap allowed for a higher number of scans per cycle time.

Acquired MS/MS spectra for high ranking PLS-R model features were compared to experimental and theoretical data accessed from the Human Metabolome Database (HMDB) and MassBank of North America (MoNA). Compound Discoverer in conjunction with a molecular formula calculator was used to propose potential molecular formulas for MS features of interest.

### 2.6 Solid Phase Extraction of Blue Crab Urine, Extract Preparation, and ^1^H NMR and 2D NMR Spectral Acquisition of Urine Extract

One urine sample (Sample A) collected from blue crabs that were fed oysters was selected for extraction and further NMR spectral analysis as it had the second highest relative concentration of^1^ H NMR signals for the metabolites of interest, identified after normalizing ^1^H NMR TMSP peak intensities across samples, and also had sufficient volume available for further analysis. The sample with the highest relative concentration of the desired ^1^H NMR signals (Sample B) was not used because there was an insufficient volume of urine available for continued spectral analysis. It was maintained for final analysis discussed later in Section 2.10. Metabolites from urine sample A were concentrated by solid phase extraction (SPE) using an ENVI-18 SPE column (3 mL; 0.5 g). The Solid Phase Extraction SPE cartridge was attached upright to a vacuum manifold, primed with 1 mL LC-MS grade methanol, washed with 1 mL deionized H_2_O. A 1 mL aliquot of urine was loaded onto the column, which was then washed with 1 mL deionized H_2_O, and 1 mL LC-MS grade methanol was used to elute metabolites from the column. This eluate was portioned into two vials (90% for NMR spectral analysis; 10% for future LC-MS or other analysis), the solvent removed *in vacuo*, and the extracts stored at – 20 °C. The extract assigned to NMR spectral analysis was dissolved in 128 μL D_2_O and combined with 46 μL phosphate buffer (0.1 M in D_2_O, pH 7.4), 16 μL TMSP internal standard, and vortexed for 5 min. Then, 175 μL of this mixture was transferred via micropipette to a 3 mm × 178 mm NMR tube (Norell Sample Vault Series).

A Bruker Avance HD 800 MHz NMR spectrometer with a 3 mm cryoprobe was used to acquire NMR and 2D NMR spectra of desalted urine extracts. ^1^H NMR spectra were acquired in 128 scans using the ZGESGP pulse sequence. Homonuclear correlation spectroscopy (COSY) spectra were obtained with 32 scans using a phase-sensitive Double Quantum Filtered COSY pulse sequence with Excitation Sculpting using 180 water-selective pulse and trim pulses (cosydfesgpphpp). Heteronuclear single quantum coherence (HSQC) spectra were collected with 180 scans in phase-sensitive mode with echo-antiecho and adiabatic pulses for inversion pulse sequence (hsqcetgpsp). Heteronuclear Multiple Bond Correlation (HMBC) spectra were acquired with 300 scans using a magnitude-mode presaturated pulse program with low-pass J-filter (hmbcgplpndqf).

### 2.7 NMR Spectral Processing and Interpretation for Urine Extract

Bruker TopSpin 4.3.0 was used to process and analyze ^1^H NMR spectra, which were calibrated by setting the TMSP peak to 0.0 ppm and manually phased. The chemical complexity of blue crab urine extracts created signal overlap that prevented the assignment of individual proton signals to particular metabolites. As such, ^1^H NMR spectral data were used to exclude particular candidate structures from consideration when experimental or predicted ^1^H NMR spectral features were absent from urine extract spectra.

### 2.8 Identification of Metabolites from NMR Spectroscopic and LC-MS Data via Database Investigation and Use of Authentic Chemical Standards

Molecular formulas (Table 2) and MS^2^ spectral peaks were searched in publicly available databases. Prioritization for annotation was given to molecular ion *m*/*z* 155.0816 as it had the highest VIP Score across all potential metabolites of interest and appeared in all four of the PLS-R models, indicating it may contribute more to the fear response in oysters than other metabolites in blue crab urine. Initial searches were conducted using accessible databases in Compound Discoverer: MZCloud, Kegg, and ChemSpider. Additional databases compatible with Compound Discoverer were identified, imported, and searched including: LipidBlast-VS68-Neg, LipidBlast-VS68-Pos, EFS_Library, EFS_HRAM_Spectra_Library. Additional libraries, including MassBank, Global Natural Products Social Molecular Network (GNPS), and the National Institute of Standards and Technology (NIST), were searched manually. Manual searches were conducted with a 3 ppm mass error tolerance. Additional details leading to the partially annotated features are found in Cepeda^42^ and Jaco.^43^

SIRIUS & CANOPUS (version 5.8.3)^44,45^ were used to propose candidate structures that were potentially not included in spectral libraries. The mass spectral and tandem mass spectral data were exported as MZML files and then imported into SIRIUS to predict molecular formulas and candidate molecular structures using CSI: FingerID. This machine learning model was used to predict molecular properties from MS^2^ data, evaluate the similarity of the unknown compound to public databases, and generate a list of possible molecular structures under the following parameters: Isotope pattern filtering, 3 ppm MS^2^ mass accuracy. MS^2^ isotopes were scored, and the following additional databases were searched during computation.: Bio Database, Biocyc, CHEBI, COCONUT, EcoCycMine, GNPS, HMDB, HSDB, KEGG, KEGG Mine, KNApSAcK, Maconda, MeSH, NORMAN, Natural Products, Plantcyc, PubChem, PubMed, YMDB, YMDB Mine, and ZINC bio. Resultant database hits were prioritized if all the observed fragments matched database entries and if SIRIUS structural prediction scores were above 70%. Lower-priority candidate structures that did not meet these criteria were not pursued with authentic standards.

^1^H NMR chemical shifts were predicted for each of the candidate structures associated with the molecular ion *m*/*z* 155.0816 including 3-(1-methyl-*1H*-imidazol-4-yl)­propanoic acid (**3**), 3-(1-methyl-*1H*-imidazol-2-yl)­propanoic acid (**4)**, 3-(1-methyl-*1H*-imidazol-5-yl)­propanoic acid (**5**), 3-(5-oxo-4,5-dihydro-*1H*-pyrrol-2yl)­propenamide (**6**), 3-(1-methyl-*1H*-pyrazol-5-yl)­propanoic acid (**7**), and 4-(*1H*-pyrazol-1-yl)­butanoic acid (**8**) using ChemDraw and NMRium^46^ (Figure 6). Candidate structures were downgraded in priority if there was no ^1^H NMR signal within ± 0.15 ppm of observed signals in blue crab urine or urine extract ^1^H NMR spectra. Using 2D NMR (COSY, HSQC, HMBC) spectra of blue crab urine extract, candidate structure coupling (^1^H -^1^H, ^1^H -^13^C, and 2-to-3 bond ^1^H -^13^C) assignments were attempted. However, this was not feasible for complete structural assignment due to a considerable signal overlap in the chemically complex sample. For further analysis, standards for **3** (BLD Pharmtech Co.), **4** (aaBlocks Inc.), **5** (Enamine), **7** (Chembridge), and **8** (Alfa Chemistry) were purchased and prepared for ^1^H NMR under the same conditions listed in Section 2.10.

Once the identity of the metabolite with [M + H]^+^ *m*/*z* 155.0816 was narrowed down to a small number of candidates, its approximate concentration in blue crab urine was assessed using MS by comparing the feature’s molecular ion peak area to the peak area of serially diluted standards using methods described in Section 2.10. In order to exclude from consideration compounds whose commercial standards did not match UHPLC retention time data, individual aliquots of a blue crab urine sample were each spiked with a single chemical standard or multiple standards in combination, at concentration(s) similar to that of the metabolite’s expected concentration in blue crab urine. Spiked urine samples were analyzed via UHPLC-MS using a Vanquish UHPLC coupled to an Orbitrap ID-X Tribrid mass spectrometer under the same column conditions as described in Section 2.3.2 above. Slight variations were made to the HESI settings to better capture the fragmentation of the ion of interest and are as follows: positive ionization mode, spray voltage of 2.3 kV, sweep gas flows of 40, 8, and 1 L/h, with a vaporizer temperature of 257 °C. Data were acquired for ions with *m*/*z* 40–178.6 at a resolution of 60,000. Targeted tandem mass spectral data was acquired for *m*/*z* 155.0816 in the ion trap with higher collision energy collisional dissociation (80 eV). Molecular ion retention times and peak overlap were used to assess chemical standards coeluting with the feature of interest.

### 2.9 MS Sample Preparation and Analysis for Metabolite Quantification

Metabolite concentrations in blue crab urine were determined by comparing molecular ion peak areas (*m*/*z* 155.0816) from MS spectra to a calibration curve generated using commercial standards of candidate metabolites. MS and MS^2^ data for standards as well as for the blue crab urine sample B, which contained the highest concentration of this metabolite, were collected using the methods described in Section 2.10. For standards **3, 4,** and **5**, a serial dilution with a 1:10 dilution factor was prepared using MeOH for dilution resulting in final concentrations of 11 nM to 1.1 μM, 10 nM to 1.0 μM, and 10 μM to 1.0 μM.

The *m*/*z* 155.0816 molecular ion peak area for each injected sample was integrated using Freestyle software.^47^ Peak areas and concentration were log-transformed and the relationship fitted via linear regression in GraphPad Prizm 10.^48^ The limit of detection was 0.07 nM, corresponding to the minimum concentration necessary for peak heights above the peak detection signal-to-noise (S/N) threshold of 1.5. The resulting linear function was used to interpolate metabolite concentrations in a 9:1 (v/v) MeOH and urine sample B for this molecular ion. The calculated concentration in the diluted sample was multiplied by 10 to determine the concentration in the undiluted sample. Metabolite concentrations in other samples were calculated relative to the selected sample using molecular ion intensity for MS data initially acquired as described in Section 2.3.2. Concentration mean and standard deviation were calculated for each calibration curve, ensuring accurate quantification of the metabolite across all samples.

### 2.10 Oyster Shell Strengthening Assay to Assess Candidate Chemical Cues

We performed a second oyster bioassay summer 2024 to determine if the putative metabolites identified in the above chemical analyses do indeed induce shell strengthening in oysters. The general assay design was the same as presented earlier in Section 2.2. Oyster spat were exposed to negative and positive controls of filtered seawater and predator water as well as chemical cues in crab urine known to induce oyster shell strengthening (homarine (**1**) and trigonelline (**2**)^36^), along with the newly identified cues from this investigation. These four new cue treatments encompassed a pure solution of a molecule that is naturally found in the urine of blue crabs **(3)**, two pure solutions of two different isomers of this molecule that are not found in crab urine **(4** and **5)**, and a mixture of the natural new urine molecule as well as homarine and trigonelline at concentrations these molecules would normally be in the urine to represent a more comprehensive urine mixture (**1**+**2**+**3**; Table 1). These seven treatments were conducted with eight replicate aquaria each containing numerous oyster spat that were exposed to cues and assayed in the same manner as first bioassay with the exception that 11–32 spat were evaluated per aquaria (1,502 spat total). Four aquaria with <11 surviving spat were not included in the analysis as this low survivorship suggests poor conditions in these replicates that might affect oyster growth or ability to respond to chemical cues (three aquaria from the positive control and one from molecule **3**). Shell strength across the different cue treatments was analyzed in the same manner as in Section 2.2 using a GLMM with a gamma distribution followed by post hoc pairwise comparisons utilizing the R packages “lme4” and “multcomp” (Table 1).

**Table 1:** Oyster Shell Strengthening Assay Treatments and Concentrations, Using Individual and Combinations of Commercial Standards Including 3-(1-Methyl-*1H*-Imidazol-4-Yl)­propanoic Acid (**3**), 3-(1-Methyl-*1H*-Imidazol-2-Yl)­propanoic Acid (**4)**, 3-(1-Methyl-*1H*-Imidazol-5-Yl)­propanoic Acid (**5**), as Well as Homarine (**1**) and Trigonelline (**2**)

Abbreviation	Treatment and Concentrations
**SW**	Natural settled seawater
**PW**	Predator water: 1.5 L natural seawater +0.5 L blue crab tank water
**1** + **2**	**1** (6.9 nM) + **2** (18 nM)
**3**	**3** (8.6 nM)
**4**	**4** (8.6 nM)
**5**	**5** (8.6 nM)
**1 + 2 + 3**	**1** (6.9 nM) + **2** (18 nM) + **3** (8.6 nM)

## 3 Results

### 3.1 Predator Diet Does Not Influence Oyster Perception of Predation Risk

Oyster spat grew stronger shells when exposed to blue crab urine without regard to predator diet (Figure 1), despite the fact that blue crab urine from predators fed different diets is chemically distinct (Figure 2). Analysis of ^1^H NMR and LC-MS data from blue crab urine revealed it is a chemically rich and complex blend composed of at least several hundred metabolites (Figure 3). PCA of both ^1^H NMR and LC-MS spectra of blue crab urine indicated substantial and statistically significant diet-dependent differences (Figure 2). Therefore, unsurprisingly, and in line with previous results,^13^ blue crab urine is chemically distinguishable based on diet (Figure 2), with the known urinary metabolites homarine (**1**) and trigonelline (**2**) contributing to this separation, as they were identified in the PC 1 loadings plot for the ^1^H NMR spectral analysis (Figure S1). Compounds **1** and **2** have been shown to play a role in oyster shell strengthening^36^ and figure predominantly in distinguishing predator diet.

**Figure 2:**
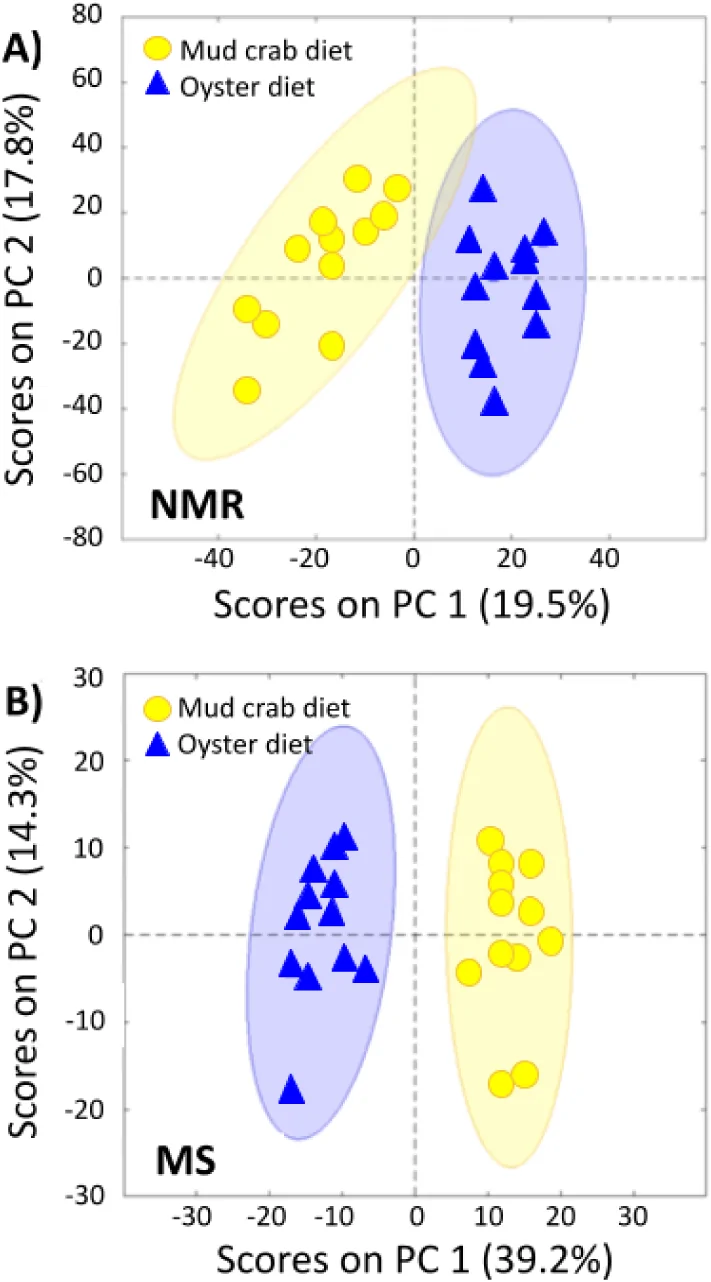
Principal component analyses (PCA) of ^1^H NMR (A) and LC-MS (B) blue crab urine metabolomics data sets, from spectral data acquired from blue crab urine fed mud crabs (*n* = 12 independent samples) and oysters (*n* = 12). Strong separation of treatments for both models indicates distinct chemical profiles for urine of blue crabs fed different diets. The first principal component axis, PC 1, accounted for 19.5% and 39.2% of the variance of the ^1^H NMR and LC-MS spectra, respectively.

**Figure 3:**
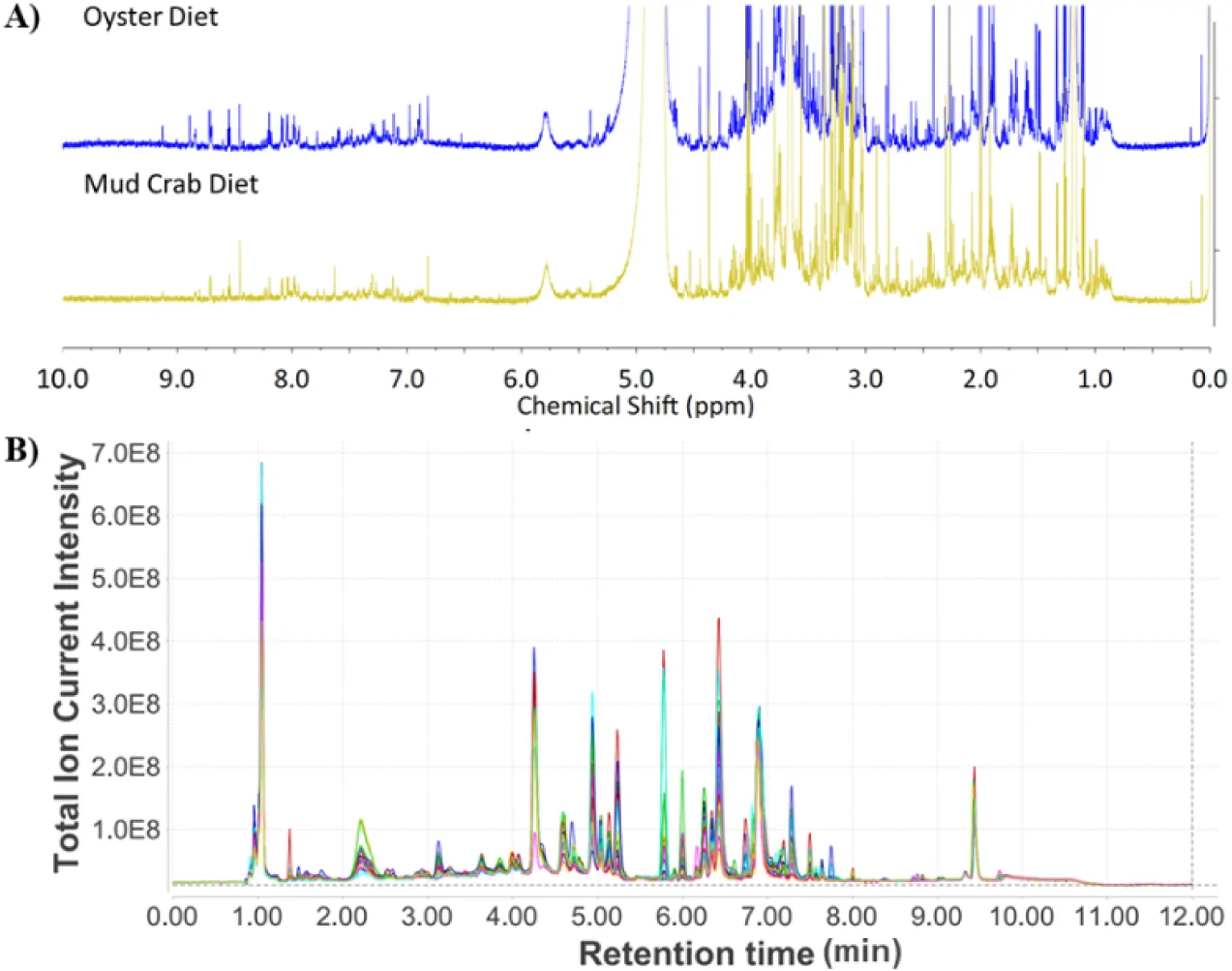
Representative ^1^H NMR spectra for oyster diet (blue spectrum) and mud crab diet (gold spectrum) blue crab urine (A) and overlaid LC-MS chromatograms from the urine of blue crabs fed mud crabs (*n* = 12) and blue crab fed oysters (*n* = 12) (B) reveal blue crab urine is chemically complex, regardless of diet, and contains a diverse array of distinct metabolites.

Despite evident metabolic differences in urine from blue crabs fed different diets, PLS-R analyses emphasized the lack of diet-dependent differences in oyster responses. Urine of blue crabs fed oysters was not more or less potent as a cue to oyster spat than urine of blue crabs fed mud crabs (Figure S2). Under both diets, the urine varied similarly in potency toward oyster spat and their chemical features associated with greater potency overlapped substantially according to PLS-R analysis (Figure S2). Thus, the molecules defining the diet-related clusters such as **1** and **2** cannot by themselves explain urine potency to oysters, since urine samples from both diets overlapped in their predicted potency based on PLS-R models. In addition, urine potency did not correlate with total concentration of urine metabolites (i.e., derived from the overall abundance of measurable chemical signals for each sample), rejecting the hypothesis that the most concentrated or chemically rich urine was most potent in fear-inducing assays. In addition, no single individual metabolite on its own significantly accounted for the fear-inducing properties of blue crab urine, as revealed by Pearson correlation coefficients (data not shown).

### 3.2 Blue Crab Urine Biomarkers Indicative of Predation Risk

Compounds **1** and **2**, previously identified as affecting oyster shell strengthening, did not explain blue crab urine potency, and so the LC-MS-based PLS-R models were further explored in order to identify novel metabolite biomarkers of predator risk. Original PLS-R models generated from LC-MS metabolomics data (Figure S2–B, R^2^ (CV) = 0.01) were improved using variable selection, since by this method, individual features are considered independent of each other. Genetic algorithm variable selection of MS features improved cross validated PLS-R models predicting oyster shell strengthening (Figure 4; R^2^ (CV) = 0.89), and on average selecting a subset of MS variables representing approximately 8% of the total. These models pinpointed 16 specific features in blue crab urine that serve as biomarkers for increased oyster shell strength (Table 2). Proposed molecular formulas for these spectral features were indicative of small nitrogenous compounds and organic acids (Table 2).

**Figure 4:**
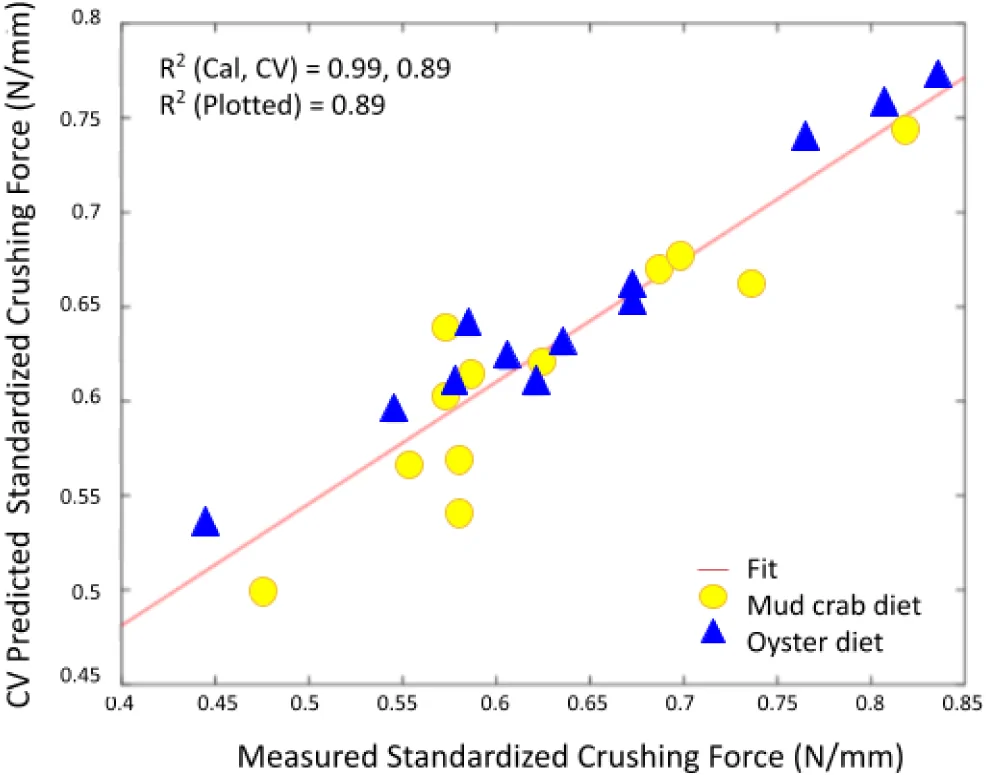
Partial least-squares regression (PLS-R) models following the application of a genetic algorithm for the oyster shell strengthening bioassay indicate a subset of metabolites in predator urine have a stronger correlation to the respective response of each prey than previously seen when considering all metabolites in blue crab urine (Figure S2–B, *n* = 12 per treatment). Only one of the final four models is shown. Plotted lines of best fit are for the LC-MS metabolomics cross-validated models (R^2^ = 0.89).

**Table 2:** Sixteen Positive Ion Mode LC-MS Features That Correlated with Increased Oyster Shell Strength^a^

Feature ([M + H] ^+^ *m/z)*	Retention Time (min)	VIP Score	Total Number of Models in Which Feature is present	Proposed Molecular Formula
155.0816	4.97	3.32	4	C_7_H_10_N_2_O_2_
392.1602	1.55	2.38	4	C_22_H_21_N_3_O_4_
276.1264	4.39	1.55	4	C_12_H_21_NO_4_S
230.1499	7.09	2.05	3	C_10_H_19_N_3_O_3_
257.1860	4.88	0.80	3	C_13_H_24_N_2_O_3_
269.1384	1.02	1.53	2	C_14_H_20_O_5_
243.1340	4.81	1.50	2	C_11_H_18_N_2_O_4_
183.1105**	7.07	0.78	2	C_7_H_16_N_2_O_2_
214.0686**	1.40	1.83	1	C_7_H_13_NO_5_
309.1379	6.21	1.79	1	C_14_H_20_N_4_O_2_S
234.1336	5.16	1.43	1	C_10_H_19_NO_5_
250.0379	1.46	1.06	1	C_8_H_11_NO_6_S
276.1078	2.48	0.96	1	Molecular Formula Uncertain
229.1545	6.87	0.96	1	C_11_H_20_N_2_O_3_
173.0922	7.16	0.86	1	C_7_H_12_N_2_O_3_
176.1282	5.30	0.84	1	C_8_H_17_NO_3_

a A list of 78 features was compiled by cross-comparing four PLS-R models generated for the oyster shell strengthening assay. Features are ordered by their VIP score relative to the model in Figure 4 and the number of models in which they were statistically significant. Metabolites marked with ** most likely contain a Na^+^ adduct.

### 3.3 Evaluation of SPE-Extracted Urine ^1^H NMR Spectra to Support Structure Elucidation

When reversed-phase SPE was applied to blue crab urine to mitigate the low sensitivity of NMR spectroscopy, the signal-to-noise ratio of the ^1^H NMR spectrum was enhanced by a factor of 3 relative to raw urine while preserving critical ^1^H NMR signals (Figure S3). Due to the inherent chemical complexity of blue crab urine, substantial ^1^H NMR signal overlap was observed, precluding resolution of peak shape and accurate *J*-coupling measurements for individual signals. Consequently, the ^1^H NMR spectrum of a representative urine extract was primarily employed to refine the list of candidate structures generated by MS annotation toward metabolite identification, based on the presence or absence of signals in the less-crowded downfield region of the spectrum, within a tolerance of ± δ 0.15, corresponding to chemical shifts predicted ^1^H NMR spectra or observed in experimental ^1^H spectra of reference standards.

### 3.4 Identification of Prioritized Metabolite

One metabolite with a high VIP score from the MS metabolomics data, signifying its important role in strengthening oyster shells in all four PLS-R models, was selected for full structure elucidation. Its molecular formula, C_7_H_10_N_2_O_2_, was a strong match for its molecular ion [M + H]^+^ *m*/*z* 155.08162 (Table 2). Database searches of its MS^2^ data led to several candidate structures composing imidazole, pyrrole, and pyrazole derivatives with differing side chains, namely: 3-(1-methyl-*1H*-imidazol-4-yl)­propanoic acid (**3**), 3-(1-methyl-*1H*-imidazol-2-yl)­propanoic acid (**4)**, 3-(1-methyl-*1H*-imidazol-5-yl)­propanoic acid (**5**), 3-(5-oxo-4,5-dihydro-*1H*-pyrrol-2-yl)­propenamide (**6**), 3-(1-methyl-*1H*-pyrazol-5-yl)­propanoic acid (**7**), and 4-(*1H*-pyrazol-1-yl)­butanoic acid (**8**) (Figure 5).

**Figure 5:**
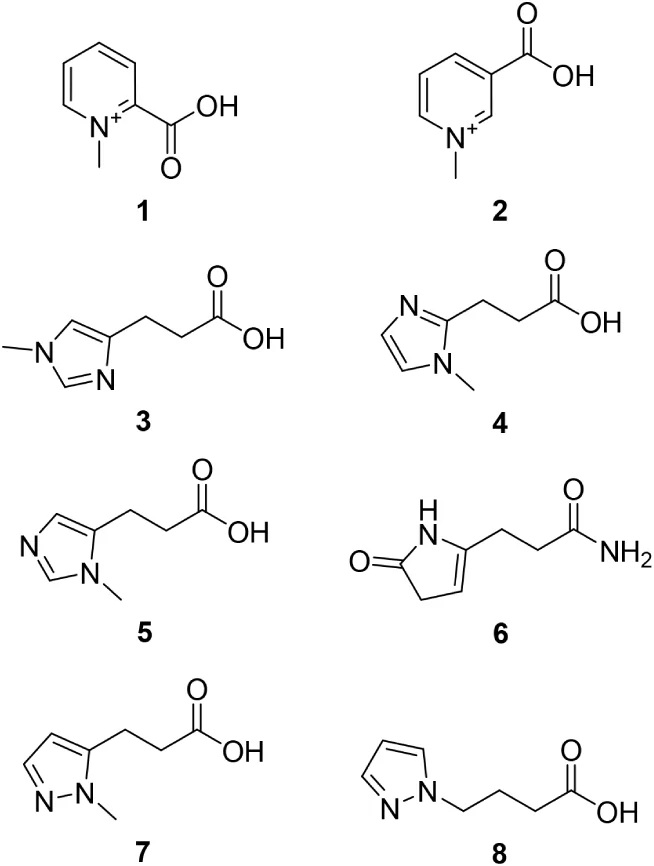
Molecular structures of blue crab urinary metabolites homarine (**1**) and trigonelline (**2**), as well as candidate compounds for the oyster shell-strengthening metabolite in blue crab urine with [M + H]^+^ *m*/*z* 155.0816:3-(1-methyl-*1H*-imidazol-4-yl)­propanoic acid (**3**), 3-(1-methyl-*1H*-imidazol-2-yl)­propanoic acid (**4)**, 3-(1-methyl-*1H*-imidazol-5-yl)­propanoic acid (**5**), 3-(5-oxo-4,5-dihydro-*1H*-pyrrol-2-yl)­propenamide (**6**), 3-(1-methyl-*1H*-pyrazol-5-yl)­propanoic acid (**7**), and 4-(*1H*-pyrazol-1-yl)­butanoic acid (**8)**.

Structures **3** and **5** seemed to be the most likely candidates for the molecular feature with *m*/*z* 155.0816, as these molecules are modified histidine derivatives frequently excreted in urine, at least in humans,^49^ and could be synthesized through the deamination and methylation of histidine through known biosynthetic pathways.^50^ However, all plausible structures (**3–8**) were retained for further consideration.

The MS^2^ fragments observed for the metabolite of interest that matched data for fragments reported in databases or by comparison with authentic chemical standards are listed in Supplementary Table S1. Candidate **6**, however, was only identified through database matching, and not predicted *in silico* (Table S1). Structures **3, 4, 5**, and **8** were predicted by *in silico* MS fragmentation as well as through empirical database matching, whereas candidate **7** was only predicted *in silico* and could only be evaluated based on its CSI:FingerID score.

Positively charged daughter ions with *m*/*z* 137.05, 109.01, and 95.01 each had a CSI:FingerID score of 100%, corresponding to a respective loss of H_2_O, CH_2_O_2_, and C_2_H_4_O_2_, consistent with candidate **7.** However, it should be noted that these fragments also matched predictions for other candidates (Table S1). Each of these candidate molecules contained a five-member heteroaromatic ring, five of which shared a C_3_N_2_H_3_ core. The presence of a methyl group on this ring structure was consistent with the fragment appearing at *m*/*z* 95.06 with a calculated molecular formula of C_5_H_6_N_2_. Furthermore, all candidates except **6** had an ethyl (**3**, **4**, **5**, **7**) or propyl (**8**) linker terminating in a carboxyl group consistent with the positive daughter ion *m*/*z* 109.076 and molecular formula C_6_H_8_N_2_ indicative of the loss of CHO_2_. Candidates **3**, **4**, **5**, **7**, and **8** differed in the location and methylation of the aromatic nitrogen. The similarities of these generated *in silico* and through database matching indicated the unknown compound was likely one of these candidates with a carboxyl group and an aromatic ring structure with a formula of C_3_H_2_N_2_.

Candidates **3–8** were examined further by comparing their predicted ^1^H NMR spectral chemical shifts to ^1^H NMR spectra of SPE-concentrated blue crab urine (sample A) (Figure S4). Due to the chemical complexity of blue crab urine, overlapping signals permeated the δ 0.7–4.5 spectral region of the urine extract ^1^H NMR spectrum, focusing attention on spectral regions downfield of δ 4.5 (Figure S3). Structure **6** was eliminated from consideration as there were no signals in the blue crab urine extract spectrum at δ 5.00 ± 0.15 for the olefinic proton predicted with nmrdb.^51,52^ Structures **7** and **8** were excluded as blue crab urinary metabolites because ^1^H NMR signals at δ 6.18 and δ 6.30 were observed in the spectra of the respective standard (Figure S4) but absent in blue crab urine and urine extract spectra (Figure S3).

Chemical standards for **3, 4,** and **5** were assessed using experimentally acquired ^1^H NMR spectra, MS^2^ fragmentation patterns, and UPLC-MS chromatographic retention times, relative to the metabolite of interest with [M + H]^+^ *m*/*z* 155.0816. Comparison of the ^1^H NMR spectrum of each standard to the well resolved aromatic region for blue crab urine extract (Figure S4) did not allow any of these standards to be eliminated as the metabolite of interest, due to similar chemical shifts and couplings. Thus, candidates **3, 4,** and **5** were further explored by comparing reversed-phase UHPLC-MS chromatographic properties of authentic standards to those of the metabolite of interest, whose retention time was 4.96 min upon initial data collection from blue crab urine. A second extracted ion with [M + H]^+^ *m*/*z* 155.0816 was observed in blue crab urine with a retention time of 7.25 min, but this ion was not identified as important to shell strengthening PLS-R models. Retention times were 5.03 (**3**), 5.39 (**4**), and 5.23 (**5**) min when analyzed individually and 5.06 (**3**), 5.42 (**4**), and 5.25 (**5**) min when spiked into blue crab urine (sample B). Candidate **3** not only had the most similar retention time to the metabolite of interest in blue crab urine but also resulted in an observed increase in peak area after spiking the urine with an authentic standard and had common MS^2^ fragmentation data (Figure S5). Upon a second UHPLC-MS analysis, the unknown feature in the blue crab urine displayed a peak at 5.05 min with an area of 2.21 × 10^5^. This peak, at approximately the same retention time (5.06 min) as **3**, showed a substantial increase in area to 4.90 × 10^5^ when a commercially acquired standard for **3** was spiked into blue crab urine. This doubling of peak area without the appearance of additional peaks confirmed that the standard of **3** coeluted with the unknown metabolite, unlike what was observed in the chromatograms of urine spiked with **4** or **5**, each of which resulted in an additional peak while the peak area of the metabolite of interest remained relatively unchanged (Figure S5). Importantly, **3** alone, when analyzed as an authentic standard, exhibited a peak area of 2.70 × 10^5^ at a nearly identical retention time (5.05 min), further validating that the increased peak area in the spiked sample can be attributed directly to the presence of **3**. Together, these results including NMR, MS, and retention time data all provided robust evidence that 3-(1-methyl-*1H*-imidazol-4-yl)­propanoic acid (**3**) is the metabolite of interest in blue crab urine, identified as having the most significant impact on oyster shell strength.

### 3.5 Natural Concentration of 3-(1-Methyl-1*H*-Imidazol-4-Yl) Propanoic Acid (**3**) in Blue Crab Urine

The natural concentration of **3**, quantified in 24 blue crab urine samples by UHPLC-MS to construct a calibration curve for the authentic standard of **3** (Figure S6), exhibited substantial variation, from below 1 nM to 1.8 μM (Table S2). This highlights the metabolic diversity within individual blue crabs or differences in physiological states, with some samples presenting trace levels below 0.03 μM, while others reached significantly higher concentrations, up to 1.8 μM. Several urine samples from blue crabs fed oysters had concentrations of **3** at or above 0.3 μM (x̅ = 0.28 **±** 0.49, Table S2), which was observed for only one urine sample from blue crabs fed mud crabs (x̅ = 0.12 **±** 0.14, Table S2), but the average concentration of **3** did not differ significantly between each diet (p = 0.67; unpaired *t* test). The observed variation of the chemical composition of urine for this compound was relatively high, with standard deviations of 0.49 μM among blue crabs fed oysters and 0.14 μM among blue crabs fed mud crabs (Table S2). The actual range of concentrations of **3** may be even greater than that observed here as each urine sample was a mixture of the urine of multiple blue crabs, and pooling the samples may have masked higher levels of variation of **3** that could exist between individual blue crabs.

### 3.6 Oyster Shell-Strengthening Bioassay Results for Candidates of Metabolite with [M + H]^+^ *m*/*z* 155.0816

Although among the candidate metabolites examined only **3** was identified as a constituent of blue crab urine, **3, 4,** and **5** were all tested in oyster shell-strengthening assays because their isomeric structures and potentially similar metabolic origins raised questions about the relationship between chemical structure and biological effect. Juvenile oysters exposed to any of **3**, **4**, and **5** grew shells that were significantly stronger, by 20–27%, than control oysters exposed only to seawater (Figure 6, Table S3). Interestingly, isomers not found in crab urine (**4** and **5**) produced statistically equivalent increases in shell strength to the urinary metabolite **3**. Each of these three compounds were similarly potent to the positive control (blue crab-conditioned water) and to **1** and **2**, previously found to induce oyster shell strengthening, albeit at much higher concentrations^29^ (Figure 6). A mixed solution of **1, 2,** and **3** produced 13% stronger shells than a solution of **3** alone at the concentration of the combined solution, but this difference was not statistically significant (coefficient estimate = 0.14, z = 1.75, *p* = 0.58).

**Figure 6:**
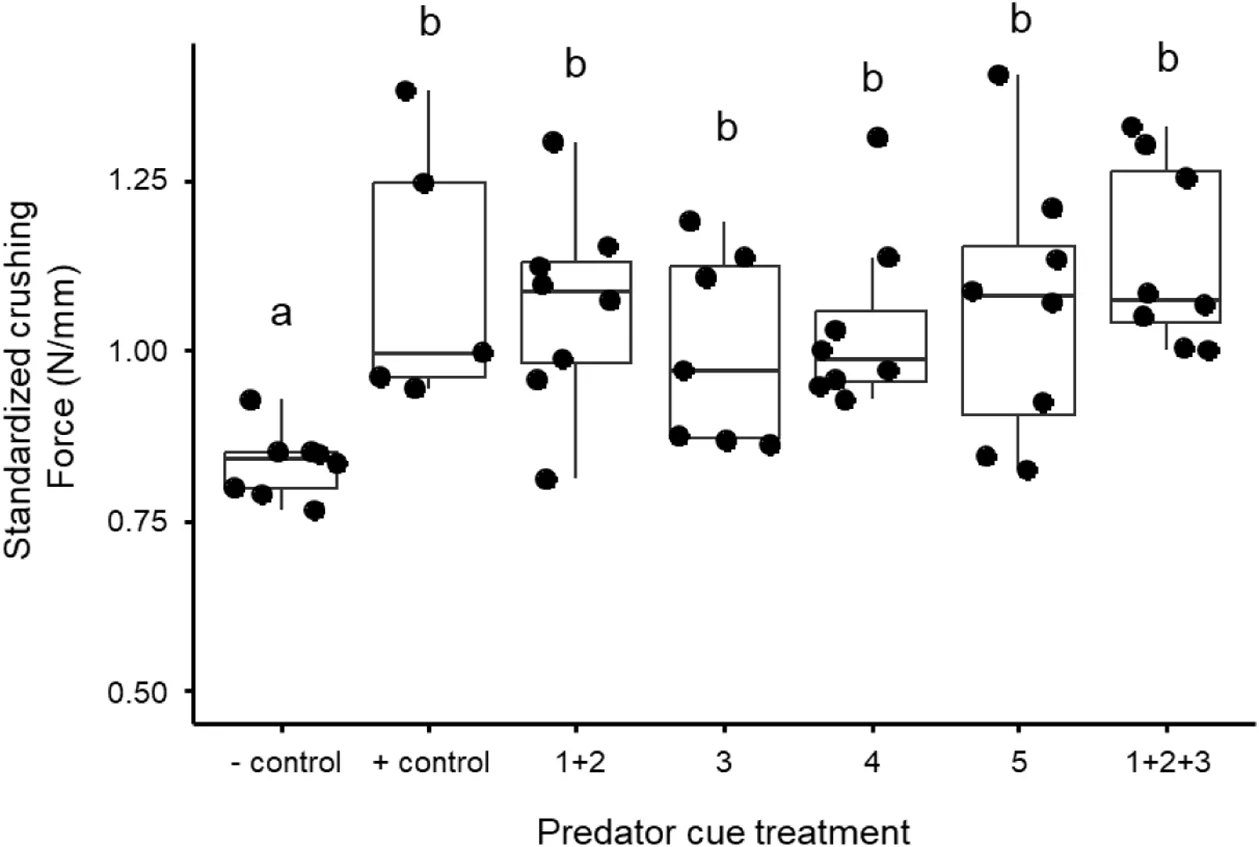
Standardized crushing force (N/mm) of oysters exposed to different predator cue solutions (- control = plain seawater, + control = water from a predator tank, **1**+**2** = solution of homarine (6.9 nM) and trigonelline (18 nM), **3** = solution of a molecule naturally found in blue crab urine (8.6 nM; 3-(1-methyl-1H-imidazol-4-yl)­propanoic acid), **4** = solution of an isomer to molecule **3** (8.6 nM; 3-(1-methyl-1H-imidazol-2-yl)­propanoic acid), **5** = solution of a second isomer to molecule **3** (8.6 nM; 3-(1-methyl-1H-imidazol-5-yl)­propanoic acid), **1**+**2**+**3** = solution of these molecules at concentrations found within crab urine at their aforementioned concentrations. Boxes indicate the 25th and 75th percentiles, whiskers denote data points no farther than 1.5x interquartile range and the median is shown by the horizontal line within each box. Black circles represent mean shell strength of oysters within a jar exposed to that particular solution (*n* = 11–32). Distinct letters above each box denote significant differences between treatments in line with Compact Letter Display convention.

Overall, we identified a metabolite, **3**, not previously known as a constituent of animal urine, contained in the urine of blue crabs fed two different diets (oysters and mud crabs). This metabolite was predicted via multivariate statistical models to play an important role in inducing juvenile oyster shell strengthening and was confirmed to do so through assays with live oyster spat at concentrations found in blue crab urine.

## 4 Discussion

In this study, metabolomics analysis of predator urine has been used to identify waterborne molecules that prey use to determine risk which enables better understanding of the mechanisms of predator–prey signaling as well as the ecology of oyster-predator interactions. This study revealed a small subset of metabolites found in urine to initiate a fear response in juvenile oysters. Although not all of the relevant molecules revealed by multivariate statistical models of metabolomics data were identified, 3-(1-methyl-1*H*-imidazol-4-yl) propanoic acid (**3**) was identified as the most important molecule inducing shell strengthening when presented at realistic environmental concentrations (8.6 nM, once diluted in seawater, Figures 4 and 6, Table 2). Importantly, this study also demonstrated the use of nontargeted metabolomics coupled with multivariate statistical modeling can determine ecologically relevant metabolites present at nanomolar concentration within chemically complex matrices such as predator urine, an approach that, to our knowledge, has rarely been used in studies of predator–prey interactions.

Despite the rich history of chemical investigations on bioactive molecules, the substances that elicit fear responses in prey remain poorly known, particularly in comparison to those compounds present in the tissue of prey that deter feeding. Unlike the molecules identified here, most feeding deterrents are specialized compounds (secondary metabolites) that span a range of configurations and functional groups, with many hundreds identified from marine organisms.^53,54^ In contrast, there are only a handful of fear-inducing compounds successfully identified from aquatic systems, with these molecules either having known physiological functions or being common metabolic byproducts. In addition to the ones we have identified here, waterborne molecules producing fear responses include bile acids released by fish that produce morphological defenses in *Daphnia* spp., aliphatic sulfates released by *Daphnia* that trigger colony formation in *Scenedesmus* algae, and copepodamides released by a variety of copepods which induce toxicity, bioluminescence, and other antipredator responses in a variety of microalgae.^19,55–58^ In terrestrial systems, alkyl pyrazine analogs are important constituents of blends used by mice^59^ and deer^60^ to sense and avoid predators. Rodents exhibit avoidance behavior in the presence of phenylethylamine, a metabolite prevalent in the urine of many carnivores.^21^

Knowing the identity of compounds that drive specific responses in predators and prey is essential to address fundamental questions regarding sensing systems and ecological interactions based on perception of chemical cues and signals. For instance, the successful identification of feeding deterrents provides insights into prey-predator evolution and can help explain population patterns and species diversity.^61^ The extent to which we can address such questions in the context of prey risk responses to predators will depend on the continuing identification of molecules that determine prey responses. Similarly, identifying the specific metabolites that induce fear can also establish the framework for studying lesser-known chemoreceptors, which in turn would offer insight into the evolution of sensor specificity and the metabolic pathways relevant to predator–prey relationships.

The metabolite found to be most influential in oyster shell strengthening was identified as **3**, whose structure was elucidated by LC-MS and ^1^H NMR spectroscopy, followed by validation of chromatographic and spectroscopic properties with a commercial standard (Figures 5, 6, and S4, Table S4). Although there is limited evidence relating to the biosynthetic origin of this compound, it appears to be a derivative of histidine, an essential amino acid whose downstream metabolites are recognized in mammalian systems as contributors to vascular, metabolic, and neurological health.^62,63^ Microbiota in human and mouse guts have been found to convert histidine into derivatives such as imidazole propionate via deamination and reduction enzymes.^64,65^ If similar enzymes are present in the digestive systems of marine invertebrates, such as blue crabs, the methylation of imidazole propionate via an *N*-methyltransferase could serve as a potential biosynthetic origin for **3**. In future, this hypothesis could further be explored through proteomic profiling of crustacean gut tissue to identify, for example, histidine-modifying enzymes.

The present study identifying **3** as an important fear-inducing metabolite, along with the previous identification of **1** and **2** playing similar roles but at higher concentrations,^18,36^ suggests that low molecular weight nitrogen-containing cyclic compounds are important for oysters’ perception of danger. Proposed molecular formulas (Table 2) for LC-MS features important in PLS-R models in this study (Figure 4) also suggest that additional fear cues important to oysters are polar nitrogen and oxygen-containing molecules that remain to be fully characterized. Given the large number of metabolites in urine, such as the many hundreds observed in blue crab urine here, this convergence on a small number of structural types as fear cues is surprising. Interestingly, exposure to either **4** or **5**, neither of which naturally occur in blue crab urine, but both of which are structurally similar to **3**, elicited similar defense responses from oysters compared to **3** (Figure 6). Thus, low-cost isomers of **3** may be helpful in the manipulation of prey responses, offering a new strategy to establish strong oyster reefs for long-term aquaculture and conservation efforts.

Metabolomics methods are particularly well suited to identifying the metabolites involved in conveying risk to prey because they involve a holistic examination of potential chemical cues. Urine is complex; the LC-MS analysis in the current study revealed 712 features, most of which are expected to be unique metabolites, and pinpointed 16 metabolites potentially responsible for triggering fear in oysters (Table 2). More traditional methods of identifying bioactive molecules depend on sequential separation of the original mixture into more refined fractions, each of which contains only a subset of the original metabolome. The problem incurred by separating blends into individual chemical components, each of which plays a role in biological activity, is magnified if blend components require particular ratios or concentrations to be salient. Thus, bioassay-guided fractionation can fail in cases in which many compounds are important for a biological effect, as is true for oyster responses to chemical cues. In the current study, **3** was the most important, but not the only, metabolite in blue crab urine to result in oyster shell strengthening. Even when **3** was present at concentrations too low to presumably induce oyster defenses, other urinary metabolites such as **1** and **2** (Table S4) (as well as other unidentified metabolites in Table 2) could result in robust responses by juvenile oysters. The multivariate statistical analysis of metabolomics data, bolstered by genetic algorithms to prioritize the most important features, enables identification of such subtleties in biological responses to complex mixtures.

Metabolomics tools for discovery of chemical cues also takes advantage of correlations between potency and chemical constituents of blends, using many independently harvested samples whose chemical composition and biological activities vary. Individual variation in the concentration of bioactive metabolites is unsurprising; animal metabolism and urine composition are influenced by age, sex, diet, genetics, and environmental conditions.^21,26^ External stressors such as climate change and exposure to anthropogenic contaminants (i.e., metals, plastics, and pollutants) also impact animal metabolism. Some of these factors almost certainly affect metabolites released in the urine of blue crabs, where the concentration of important metabolites and in turn, prey perception of risk, varies (Figures S2 and S 4, Table S4). As noted, the natural concentration of **3** across 24 independently collected blue crab urine samples measured between below the detection limit (0.07 μM) to 1.80 μM, with an average of 0.15 μM across blue crabs fed two different diets (Table S2). The importance of this variation was revealed by the variation in potency of different urine samples in oyster responses. Such variation in the concentration of bioactive molecules can make it difficult to determine the key molecules unless accounted for in the experimental design. Metabolomics methods capitalize on this variation by explicitly correlating chemical features with biological potency and using statistical models to predict those features associated with potency.

Even with a metabolomics approach, the sheer number of molecules in urine makes it challenging to elucidate the individual metabolites responsible for bioactivity. However, variable selection applied to metabolomics data sets enables models to be generated from a subset of cues that are most biologically relevant.^66,67^ Genetic algorithm variable selection in the current study resulted in prioritization of fewer than 10% of the total number of over 700 features. Cross-comparison of the different models revealed a small number of candidates that were important in several models, one of which (**3**) was most important governing fear responses in oysters (Table 2). Thus, using a metabolomics approach employing cutting-edge methods for model optimization provides a viable pathway for identifying bioactive molecules even in complex mixtures.

This study adds to the growing body of evidence that prey rely on the detection of common and ubiquitous molecules to convey fear. Cueing metabolites that are regularly produced by predators is expected to be a favored strategy by prey since the production and release of such metabolites is difficult for a predator to suppress, so they present a reliable signal of risk. However, using ubiquitous metabolites as chemical cues may entail a disadvantage if such metabolites are associated not only with dangerous predators but also harmless members of the community. One would expect prey to be selected to respond most intensely to more dangerous predators, as occurs with mud crabs exposed to blue crab urine. Mud crabs, like other prey,^33,35^ respond more to metabolites (in this case **1** and **2**) that are enhanced in the urine of predators that have consumed conspecifics,^18^ presumably because this more directly signals imminent threat. However, while it is true that prey species often respond more strongly to metabolites of predators that have consumed prey conspecifics, this is not universally true, and the importance of diet remains unresolved.^34^ In the current study, oysters responded similarly to urine from blue crabs regardless of diet (Figures 1, 4, and 5), indicating that oysters differ from mud crabs in their sensitivity to this aspect of predator cues. And while the concentration of **3**, the most important indicator of predation risk to oysters, was 0.28 μM in urine of blue crabs fed oysters (vs 0.12 μM when the blue crab diet was mud crabs), the wide range of naturally occurring concentrations of **3** made this difference insignificant (Table S3). Rather, oysters responded to a variety of molecules (**1–3**) and others based on the features identified in our model (Table 2). Why this set of molecules is used by oysters to determine their risk response is unclear. It could simply reflect the fact that there are a potentially large number of molecules that could indicate risk, regardless of not strictly signaling predation on conspecifics. Alternately, detecting predators more generally might have advantages for a sessile organism where even a generalist predator that is not immediately threatening might become so in the future. Understanding how the selective pressures on prey species, or constraints on prey perception of chemical cues, determines salient signal molecules must await further studies identifying these bioactive fear molecules in other predator–prey systems.

## Supplementary Material



## Data Availability

All MS (study ID ST004912) and NMR spectral data (study ID ST004965) are publicly available on Metabolomics Workbench, Project ID PR003146, Project doi: 10.21228/M81G35 at https://dev.metabolomicsworkbench.org:22222/data/DRCCMetadata.php?Mode=Project&ProjectID=PR003146&Access=MrjM2386.

## Notes

Table S1 Comparison of final six candidate structures; Table S2 Concentrations of **3** in blue crab urine; Table S3 Statistical results from generalized linear mixed model of oyster responses; Table S4 Concentration of metabolites in blue crab urine; Figure S1 NMR spectra and loadings for PCA; Figure S2 PLS-R for NMR and MS metabolomic data; Figure S3 NMR spectra of blue crab urine before and after concentration by SPE; Figure S4 NMR spectra of commercial standards; Figure S5 Extracted ion chromatograms from blue crab urine and commercial standards; Figure S6 Calibration curve for quantification of **3** in blue crab urine (PDF)
