# Supplementary material for: Predator Cues Induce Defensive Responses in Marine Prey: A Metabolomics Decoding of Blue Crab Urine Biomarkers
Source: J Proteome Res. 2026 Aug 28;25(10):5549–61. doi: 10.1021/acs.jproteome.6c00084 (PMC13640505; doi:10.1021/acs.jproteome.6c00084)
Supplement: Supplementary file 1 [file pr6c00084_si_001.pdf]

Supporting Information

Predator Cues Induce Defensive Responses in Marine Prey: A Metabolomics Decoding of Blue Crab Urine Biomarkers

Marisa R. Cepeda,[1] *Benton R. Jaco,*[2] *Benjamin A. Belgrad,*[3] *Maria E. Branch,*[2] *Samuel G. Moore,*[1,4] *Sarah H. Roney,*[2] *David A. Gaul,*[1,4] *Delbert L. Smee,*[3,5] *Marc J. Weissburg,*[2] *Julia Kubanek*[1,2,4,*]

[1] School of Chemistry and Biochemistry, Georgia Institute of Technology, Atlanta, GA 30332, USA

[2] School of Biological Sciences, Georgia Institute of Technology, Atlanta, GA 30332, USA

[3] Dauphin Island Sea Lab, Dauphin Island, AL 36528, USA

[4] Parker H. Petit Institute for Bioengineering and Bioscience, Georgia Institute of Technology, Atlanta, GA 30332, USA

[5] School of Marine and Environmental Sciences, University of South Alabama, Mobile, AL 36608, USA

*Corresponding author: julia.kubanek@biosci.gatech.edu

Table S1: Comparison of final six candidate structures for metabolite $[M+H]^+$ *m/z* 155.0816 with chemical formula $C_7H_{10}N_2O_2$

Table S2: Concentrations of 3-(1-methyl-*1H*-imidazol-4-yl)propanoic acid **(3)** in blue crab urine from blue crabs fed either oysters or mud crabs

Table S3: Statistical results from generalized linear mixed model comparing the magnitude of oyster responses from different predator cue solutions to a negative control of natural settled seawater.

Table S4: Concentration of homarine, trigonelline, and concentrations of 3-(1-methyl-*1H*-imidazol-4-yl)propanoic acid in blue crab urine

Figure S1: ^{1}H NMR spectra and the corresponding loadings plot for the principal component analysis of the ^{1}H NMR blue crab urine metabolomics dataset

Figure S2: Partial least squares regression (PLS-R) analysis for ^{1}H NMR and MS metabolomic data relating predicted vs. observed urine potency prior to the application of the genetic algorithm

Figure S3: ^{1}H NMR spectra of a blue crab urine sample before and after concentration by solid-phase extraction

Figure S4. ^{1}H NMR spectra of commercial standards

Figure S5: Extracted ion chromatogram (EIC) for molecular ion [M+H]+ *m/z* 155.0816 from blue crab urine (BCU) and commercial standards

Figure S6: Calibration curve for quantification of 3-(1-methyl-*1H*-imidazol-4-yl)propanoic acid in blue crab urine (**3**)

Table S1. Properties of candidate structures for molecular feature $[M+H]^+$ *m/z* 155.0816 are important to the PLS-R metabolomics model of blue crab urine based on database matches and *in silico* predictions. Bolded CID fragment values indicate observed fragments that corresponded to those present in commercial standards or that were reported to public databases. For candidates supported with *in silico predictions,* CSI:FingerID score reflects structural prediction confidence (0 = exact match). The Tanimoto similarity score indicates the ratio of shared features between the predicted and known compounds, with a value of 0.85 corresponding to 85% similarity in biological activity (Jasial et al., 2016). Please note, not all *in silico* predictions had corresponding database matches and vice versa, thus explaining why some candidates do not have data for both methods of structure annotation.

Candidate Structure	Observed CID Fragmentation	Match Source(s)	CSI:FingerID Score	Tanimoto Similarity Score
**3**	**138.01**, **137.05**, 136.13, 127.06, 114.08, 113.19, 112.19, 111.22, **109.01**, 96.45, **95.01**, 82.13, 81.02, 72.95, **72.13**, **58.01**	KEGG Mine, Consistent with standard (Enamine US Inc)	-44.29	0.77
**4**	138.01, **137.05**, 136.13, 127.06, 114.08, 113.19, 112.19, 111.22, **109.01**, 96.45, **95.01**, 82.13, **81.02,** 72.95, 72.13, 58.01	PubChem, Consistent with standard	-48.48	0.72
**5**	138.01, **137.05**, 136.13, 127.06, **114.08**, 113.19, 112.19, 111.22, 109.01, **96.45**, **95.01**, 82.13, 81.02, 72.95, **72.13**, **58.01**	PubChem, Consistent with standard (BLD Pharmatech Co.)	-41.70	0.76
**6**	**138.01, 137.05, 136.13, 127.06, 114.08, 113.19, 112.19, 111.22, 109.01, 96.45, 95.01, 82.13, 81.02, 72.95, 72.13, 58.01**	Consistent with spectra reported to NIST	N/A	N/A
**7**	N/A	PubChem	-46.09	0.72

**8**	138.01, **137.05**, 136.13, 127.06, 114.08, 113.19, 112.19, 111.22, **109.01**, 96.45, 95.01, 82.13, **81.02,** 72.95, 72.13, 58.01	PubChem	-55.21	0.66

Table S2. Concentrations of 3-(1-methyl-*1H*-imidazol-4-yl)propanoic acid (**3**) in blue crab urine from blue crabs fed oysters (BCO) and blue crabs fed mud crabs (BCM), derived from UPLC-MS calibration curve with a commercial standard. The UPLC-MS calibration curve was used to calculate the concentration of **3** in blue crab urine sample B and then related to all other urine samples by comparison of the relative intensity of their molecular ion peaks from prior analyses. All concentrations, means, and standard deviations are listed µM. Samples for which the molecular ion peak height (at *m/z* 155.0816) was below the UPLC-MS signal-to-noise detection threshold (1.5) of approximately 0.07 nM are listed as zero, BCO $\bar{x} = 0.28 \pm 0.49$ µM, BCM $\bar{x} = 0.12 \pm 0.14$. Sample A in section 2.6 refers to sample BCO 3, which was used for NMR analysis, and sample B, used for determination of the concentration of **3**, was taken from sample BCO 7.

Sample ID	Concentration (µM)
BCO 1	0.00
BCO 2	0.35
BCO 3	0.40
BCO 4	0.16
BCO 5	0.00
BCO 6	0.00
BCO 7	1.80
BCO 8	0.09
BCO 9	0.15
BCO 10	0.15
BCO 11	0.28
BCO 12	0.00

Sample ID	Concentration (µM)
BCM 1	0.43
BCM 2	0.26
BCM 3	0.20
BCM 4	0.11
BCM 5	0.00
BCM 6	0.03
BCM 7	0.00
BCM 8	0.22
BCM 9	0.26
BCM 10	0.00
BCM 11	0.00
BCM 12	0.00

Table S3. Statistical results from generalized linear mixed model comparing the magnitude of oyster responses from different predator cue solutions to a negative control of natural settled seawater. Bold, italicized numbers indicate significant differences.

Cue solution	Coefficient	t – value	*p* – value
+ control	0.28	3.35	*0.0008*
**1**+**2**	0.25	3.37	*0.0007*
**3**	0.17	2.11	*0.0347*
**4**	0.22	3.04	*0.0024*
**5**	0.23	3.13	*0.0017*
**1**+**2**+**3**	0.31	4.17	*<0.0001*

Table S4. Concentration of homarine, trigonelline, and concentrations of 3-(1-methyl-*1H*-imidazol-4-yl)propanoic acid (**3**) in blue crab urine collected from blue crabs fed either oysters or mud crabs

Sample ID	Homarine (µM)	Trigonelline (µM)	3 (µM)
BCO 1	5.55	3.76	0.00
BCO 2	11.17	18.52	0.35
BCO 3	0.41	3.43	0.40
BCO 4	0.66	0.34	0.16
BCO 5	0.41	1.39	0.00
BCO 6	2.765	0.54	0.00
BCO 7	0.94	11.00	1.80
BCO 8	4.45	1.39	0.09
BCO 9	1.82	0.84	0.15
BCO 10	11.42	1.81	0.15
BCO 11	66.09	11.53	0.28
BCO 12	47.60	3.43	0.00
BCM 1	7.10	4.70	0.43
BCM 2	38.09	11.71	0.26
BCM 3	35.69	9.64	0.20
BCM 4	0.65	0.83	0.11
BCM 5	0.73	1.25	0.00
BCM 6	10.94	2.2	0.03
BCM 7	2.58	2.12	0.00
BCM 8	31.25	11.17	0.22
BCM 9	6.59	4.29	0.26
BCM 10	7.36	3.19	0.00
BCM 11	28.53	0.62	0.00
BCM 12	14.40	2.27	0.00

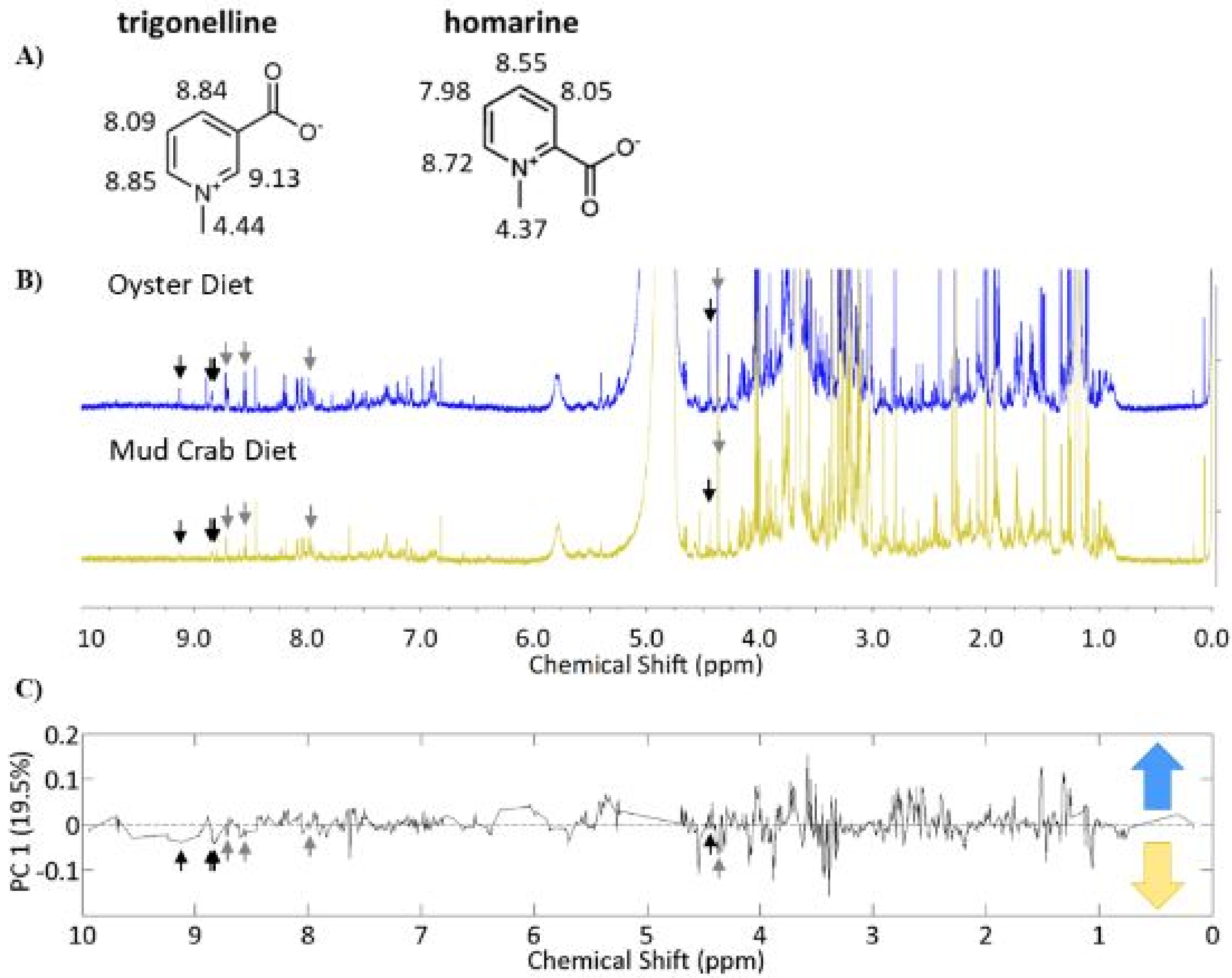

Figure S1. Trigonelline and homarine are two blue crab urine metabolites that contribute to distinct chemical profiles for blue crab urine of different diets. A) Structures of homarine (**1**) and trigonelline (**2**) with their ^{1}H NMR chemical shifts. Two representative ^{1}H NMR spectra (B) and the corresponding loadings plot (C) for the principal component analysis of the ^{1}H NMR blue crab urine metabolomics dataset. Black arrows point to peaks for trigonelline, and gray arrows point to peaks for homarine. Trigonelline and homarine peaks are negative in the loadings plot indicating that these features are enriched in the mud crab diet (yellow arrow). Features enriched in the oyster diet are positive for PC 1 in the loadings plot (blue arrow).

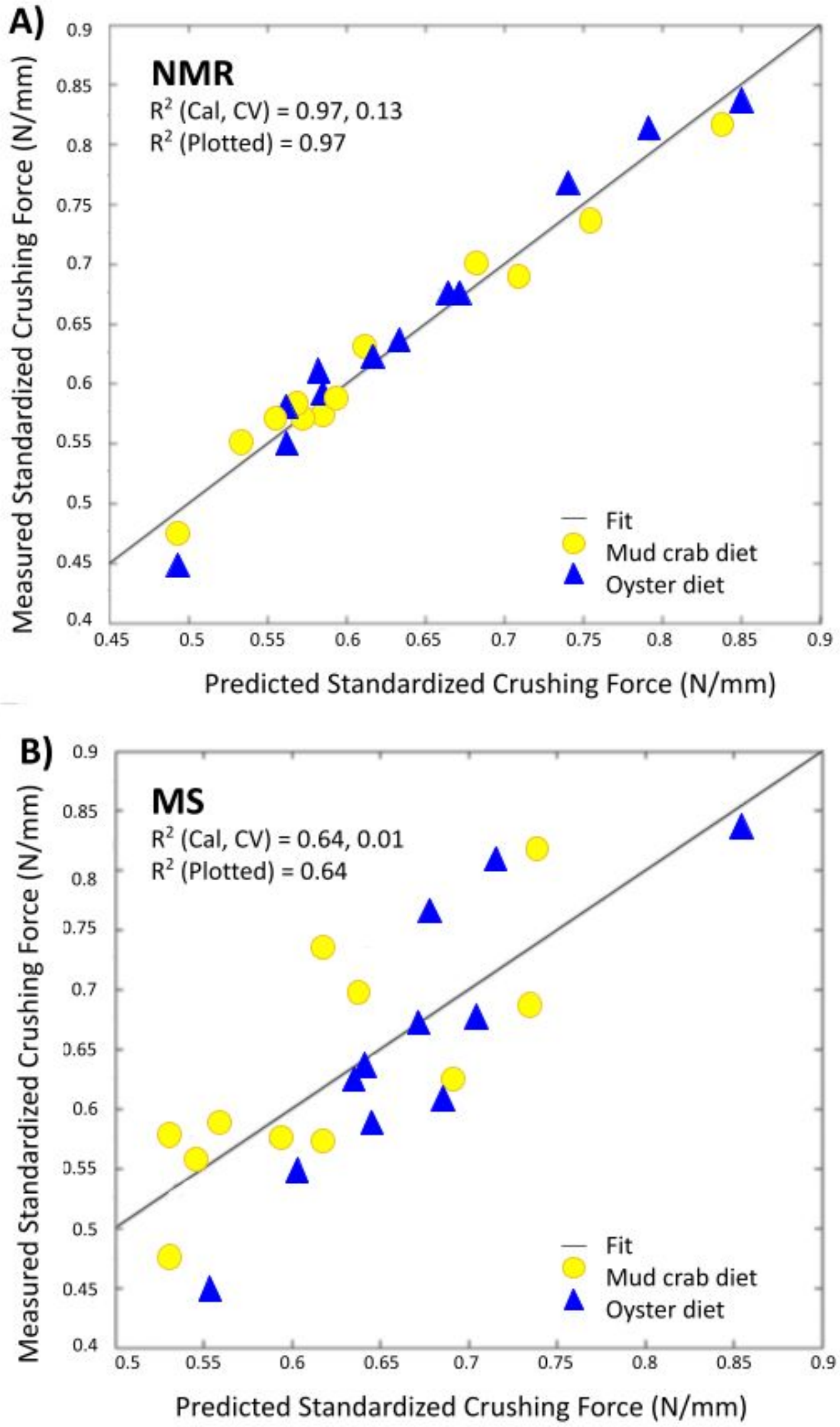

Figure S2. Partial least squares regression (PLS-R) analyses for ^{1}H NMR (A) and MS (B) metabolomics data relating predicted vs observed urine potency, showing important chemical features affecting the strength of oyster shell strengthening are not diet dependent. For both models, measured oyster shell standardized crushing force was regressed against spectroscopic chemical features to predict standardized crushing force values for each urine sample. Lines of

best fit for the ^{1}H NMR and MS metabolomics models have R^2 values of 0.97 and 0.64 respectively. Models represent the data prior to the application of the genetic algorithm (Fig. 4). These analyses are based on n = 12 urine samples for each treatment.

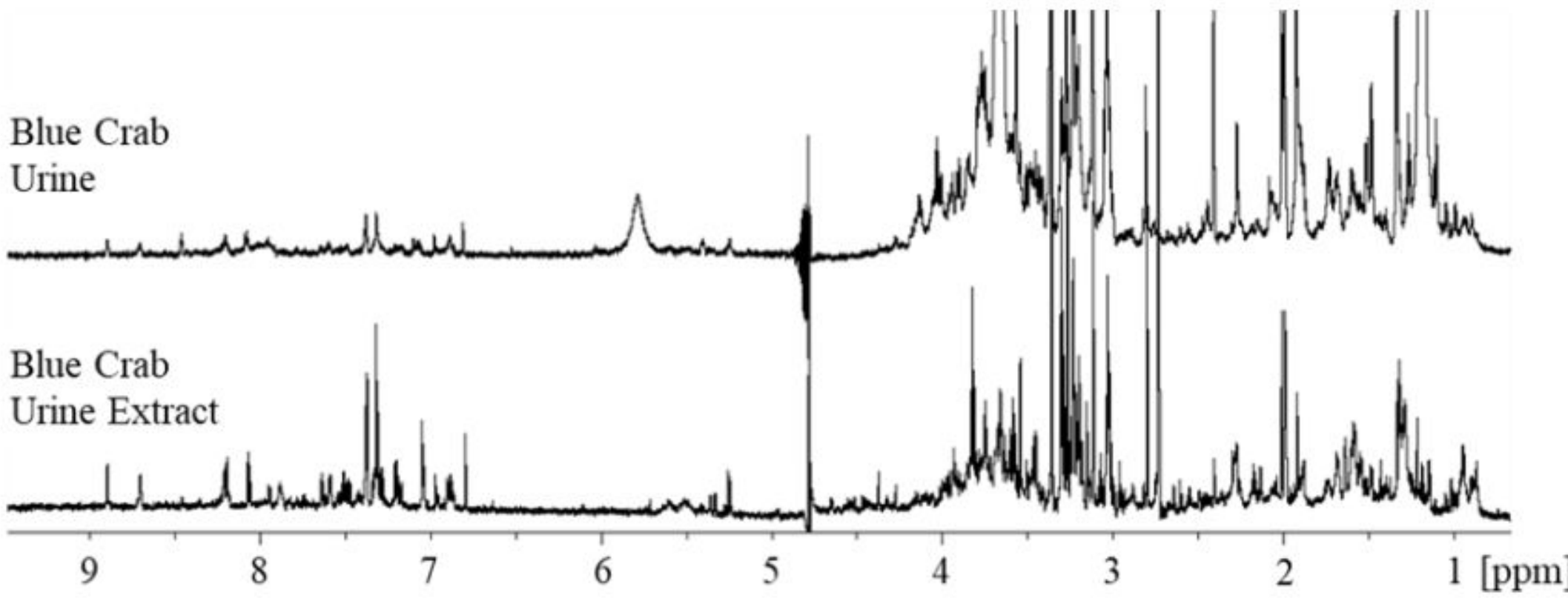

Figure S3. ^{1}H NMR spectra of a blue crab urine (sample A) before (top) and after (bottom) concentration by solid-phase extraction (SPE) (800 MHz, in buffered D_2O with water suppression).

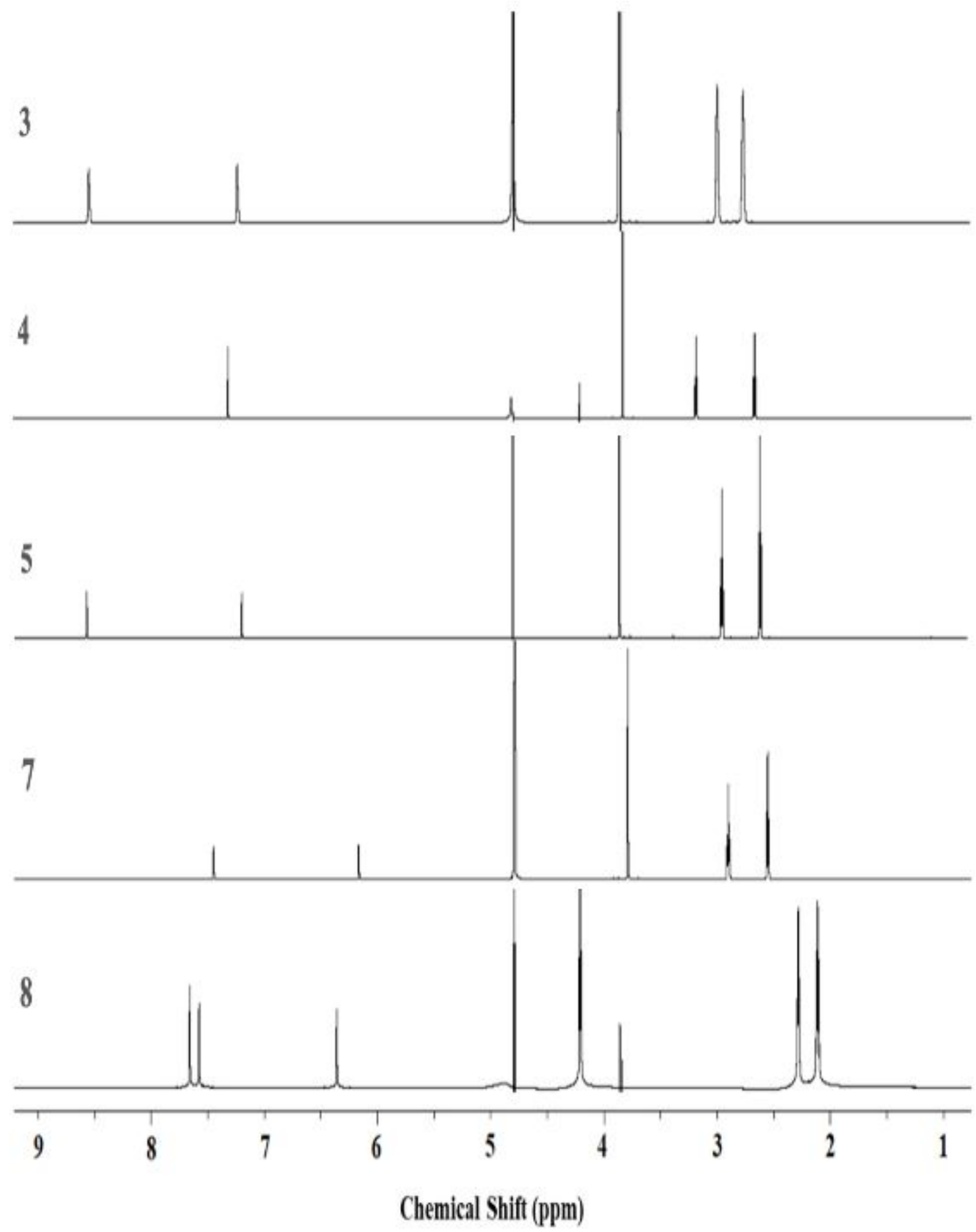

Figure S4. ^{1}H NMR spectra of commercial standards of 3-(1-methyl-*1H*-imidazol-4-yl)propanoic acid (**3**), 3-(1-methyl-*1H*-imidazol-2-yl)propanoic acid (**4**), 3-(1-methyl-*1H*-imidazol-5-yl)propanoic acid (**5**), 3-(1-methyl-*1H*-pyrazol-5-yl)propanoic acid (**7**), and 4-(*1H*-pyrazol-1-yl)butanoic acid (**8**) to compare the differences in the chemical shifts for each compound, and lead to metabolite identification. Numbers to the left of each ^{1}H NMR spectra indicate the compound associated with that spectrum.

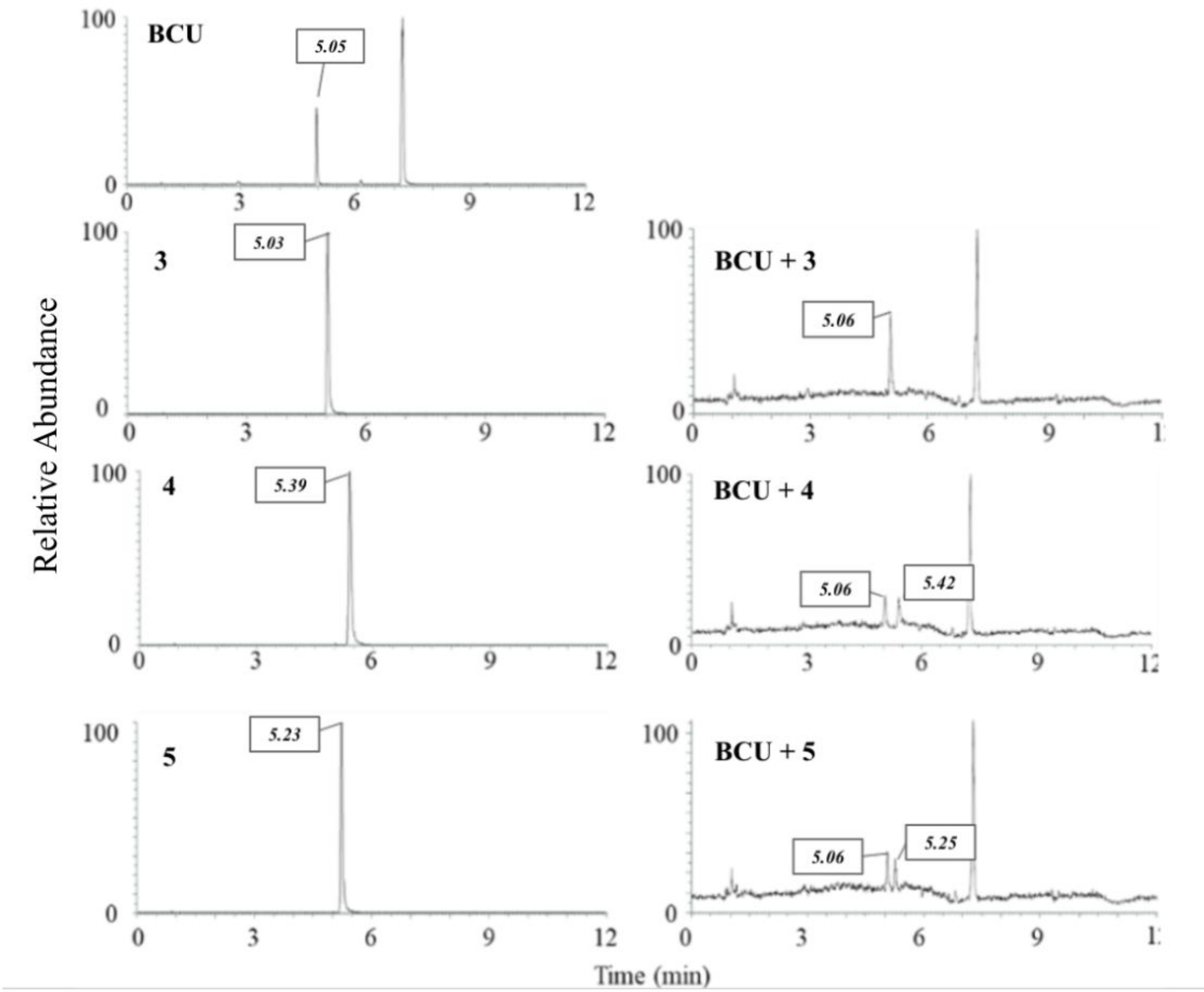

Figure S5. Extracted ion chromatogram (EIC) for molecular ion [M+H]+ *m/z* 155.0816 from blue crab urine (BCU) and commercial standards of 3-(1-methyl-*1H*-imidazol-4-yl)propanoic acid (**3**), 3-(1-methyl-*1H*-imidazol-2-yl)propanoic acid (**4**), 3-(1-methyl-*1H*-imidazol-5-yl)propanoic acid (**5**). Labels at the top left of each EIC indicate the solution: blue crab urine (BCU), **3**, **4**, and **5**; and spiked combinations (BCU + **3**, **4**, or **5**). Retention times (RT) in minutes are listed boxed and in italics for the ion peaks corresponding to the ion of interest from blue crab urine ($[M+H]^+$ *m/z* 155.0816) or chemical standards **3**, **4** and **5**.

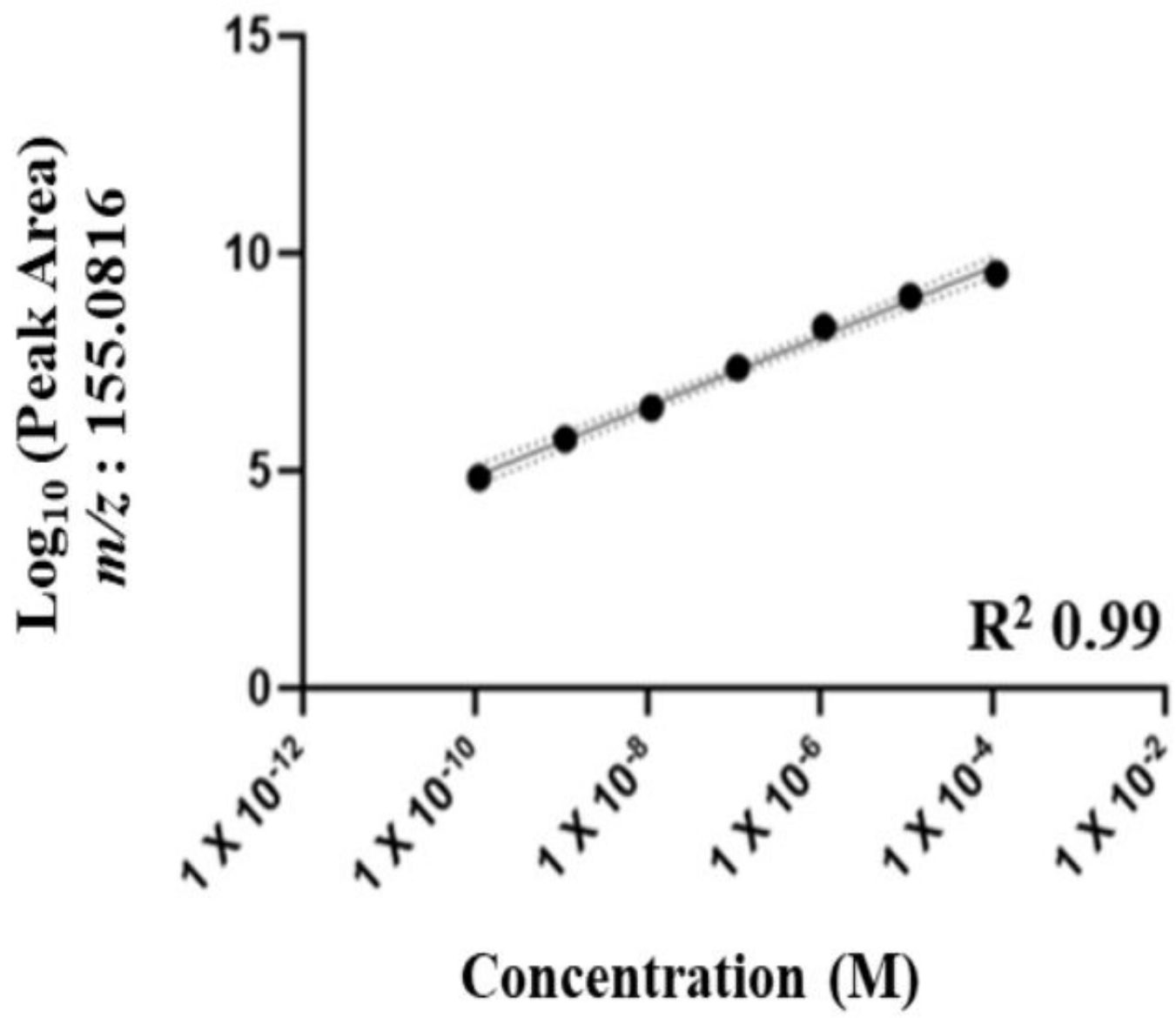

Figure S6. UPLC-MS calibration curve for quantification of 3-(1-methyl-1*H*-imidazol-4-yl)propanoic acid **(3)** in blue crab urine. Calibration coefficient (R^2) for the calibration curves indicate strong linearity across 6 orders of magnitude

References:

Jasial, S., Hu, Y., Vogt, M., & Bajorath, J. (2016). Activity-relevant similarity values for fingerprints and implications for similarity searching. *F1000Research*, *5*, Chem Inf Sci-591. https://doi.org/10.12688/f1000research.8357.2
